# *55*.*2*, a Phage T4 ORFan Gene, Encodes an Inhibitor of *Escherichia coli* Topoisomerase I and Increases Phage Fitness

**DOI:** 10.1371/journal.pone.0124309

**Published:** 2015-04-14

**Authors:** Yves Mattenberger, Filo Silva, Dominique Belin

**Affiliations:** Department of Pathology and Immunology, University of Geneva, Geneva, Switzerland; Centre National de la Recherche Scientifique, Aix-Marseille Université, FRANCE

## Abstract

Topoisomerases are enzymes that alter the topological properties of DNA. Phage T4 encodes its own topoisomerase but it can also utilize host-encoded topoisomerases. Here we characterized 55.2, a phage T4 predicted ORF of unknown function. High levels of expression of the cloned 55.2 gene are toxic in *E*. *coli*. This toxicity is suppressed either by increased topoisomerase I expression or by partial inactivation of the ATPase subunit of the DNA gyrase. Interestingly, very low-level expression of 55.2, which is non-lethal to wild type *E*. *coli*, prevents the growth of a deletion mutant of the topoisomerase I (*topA*) gene. *In vitro*, gp55.2 binds DNA and blocks specifically the relaxation of negatively supercoiled DNA by topoisomerase I. *In vivo*, expression of gp55.2 at low non-toxic levels alters the steady state DNA supercoiling of a reporter plasmid. Although 55.2 is not an essential gene, competition experiments indicate that it is required for optimal phage growth. We propose that the role of gp55.2 is to subtly modulate host topoisomerase I activity during infection to insure optimal T4 phage yield.

## Introduction

The various DNA manipulations that occur during the normal life cycle of a cell can result in three topologically altered forms of DNA: knots, catenanes, and supercoils [1]. Supercoils describe a DNA state in which the number of times the strands cross each other differs from that in an unconstrained DNA molecule. If this number is lower or higher than expected, DNA is said to be either negatively or positively supercoiled. Processes that require the unwinding or the rotation of a topologically constrained DNA double helix, such as replication or transcription, will lead to the accumulation of compensatory supercoils that hinder polymerase movement [2,3]. Although such torsional stress can be a problem, supercoiling has important physiological functions. Bacteria, normally maintain their chromosome and plasmids in a negatively supercoiled state and this is an important factor in both chromosome compaction and transcriptional regulation [4,5].

DNA’s diverse topological problems can be resolved by topoisomerases, a family of enzymes that allow DNA strands or DNA double helices to pass through each other. Type I topoisomerases transiently cut one DNA strand, releasing the superhelical tension, while type II enzymes cut both DNA strands allowing the ATP-dependent transport of one DNA double helix through the other [6,7]. Given the universality of DNA topological constraints, it is not surprising that topoisomerases are found in all three domains of life. In addition, a number of viruses, both eukaryotic and bacterial, encode their own topoisomerases, [8,9]. Because of their critical role in determining DNA topology, topoisomerases have become a target of choice for the development of antibiotics and anticancer drugs. Consequently, some type II topoisomerase inhibitors are employed clinically as potent antibacterial and antitumor agents [10,11].

*Escherichia coli* has two type I topoisomerases (Topo I and III), and two type II topoisomerases (DNA gyrase and Topo IV). The negative supercoiling of the bacterial chromosome and plasmids is homeostatically regulated by the counterbalancing activities of Topo I and Topo IV, both of which remove negative supercoils, and of DNA gyrase, which adds negative supercoils [12]. Topo III and Topo IV are primarily responsible for the unlinking of sister chromosomes during and after replication [13]. Phage T4, which infects *E*. *coli*, expresses its own topoisomerase whose three subunits are encoded by genes *39*, *52*, and *60* [14,15]. This type II topoisomerase is not absolutely essential for T4 growth, but topoisomerase mutants display the so-called DNA-delay phenotype [16–18]. Indeed, the phage topoisomerase is absolutely required for the early mode of phage DNA replication. In topoisomerase mutant phages, DNA replication depends instead on the late, recombination-dependent, join-cut-copy replication pathway (reviewed in [19–21]). In the absence of its own topoisomerase, T4 growth is completely dependent on host DNA gyrase [22].

Through the isolation of large collections of nonsense and temperature-sensitive mutants, phage T4 was the first organism in which nearly all the essential genes were identified [16,23]. In addition to the 62 essential genes, the genome was also known to encode auxiliary proteins, increasing the total number of characterized genes to 156. However, the T4 genome is predicted to have 289 protein-coding genes (open reading frames ORF); nearly all of these additional 126 genes have no known function [24]. Since most of these new unknown T4 genes have no detectable homology to any known proteins, they were considered databases orphans (ORFans). Nevertheless, many of them are present in the genomes of other related T4-like phages [25]. Since lytic phages are among the most abundant organisms on the planet [26,27], these numerous and diverse ORFans represent an enormous reservoir of unchartered genetic information, the so-called “dark matter of the biosphere” [26,28].

We previously identified several T4 ORFans whose ectopic expression significantly inhibits *E*. *coli* growth [29]. As described above, many DNA transactions require the resolution of DNA topological issues. Thus, we considered the possibility that some of the T4 ORFans might be involved in the control of phage DNA topology possibly in conjunction with the host topoisomerases. We report here on the characterization of gene *55*.*2*, a toxic T4 ORFan that affects supercoiling regulation in *E*. *coli* and whose inactivation reduces phage T4 progeny production.

## Materials and Methods

### *E*. *coli* and phage strains, plasmids and growth conditions

The bacterial strains and phage strains used in this study are listed in Table 1; the plasmids are listed in S1 File. The construction of the strains, plasmids and genomic DNA library are described S1 File. Unless otherwise stated, all *E*. *coli* strains were cultivated at 37°C in LB, M9 minimal (M9), or M63 minimal (M63) medium [30] supplemented with amino acids and carbon source as indicated. For growth on solid medium, 1.5% bacteriological agar was included. Antibiotics were used at the following concentrations: ampicillin (Ap), 200 μg ml^-1^; chloramphenicol (Cm), 30 μg ml^-1^; kanamycin (Kn), 40 μg ml^-1^; spectinomycin (Sp), 50 μg ml^-1^; and tetracycline (Tc), 7.5 μg ml^-1^.

**Table 1:** E. coli^a^ and T4 strains.

Strain	Genotype	Reference
AS1047	MG1655 *Δ lacIZYA*::*frt* pAST111	[31]
AS1050	MG1655 *Δ lacIZYA*::*frt Δ topA*::*apra* pAST111	[31]
B^E^	Prototrophic	[32]
BL21(DE3)	F^-^ *ompT gal dcm lon hsdS*_*B*_ λ(DE3)	[32]
BP199	W3110 *gyrB221*(Cou^R^) *gyrB203*(ts)	[13]
BW25113	F- *Δ(araD-araB)567 ΔlacZ4787*(::rrnB-3) *rph-1 Δ(rhaD-rhaB)568 hsdR514*	CGSC
CL001	W3110 *parE10*(ts)	[13]
CR63	F^+^, *supD*, *lamB63*	lab collection
DHB3	MC1000 *malFΔ* 3 *phoAΔ* (PvuII) *phoR*	[33]
DB503	MC4100 *malE16-1 Δ ara714*	lab collection
DB870	MG1655 *Δ ara714*, *ydeA*::*kan*	this study
DH5α	F^-^ *Δ(argF-lac)169*, φ80d*lacZ58*(ΔM15), *ΔphoA8*, *glnV44*, *deoR481*, *rfbC1*, *gyrA96*(Nal^R^), *recA1*, *endA1*, *thiE1*, *hsdR17*	CGSC
JW0204-2	BW25113 *ΔrnhA733*::*kan*	[34]
MC1000	F^-^ *araD*139 *Δ* (*araA-leu)*7697 *Δ* (*lac*)X74 *rpsL*150 *galE15 galK16 relA*1 *thi*	[35]
MC4100	F^-^ *araD*139 *Δ* (*argF-lac*)U169 *flhD*5301 *fruA*25 *relA*1 *rpsL*150 *rbsR*22 *Δ* (*fimB-fimE*)632 *deoC*1 *thi*	[36]
MG1655	F^-^ *rph-1*	CGSC
W3110	F^-^ *IN(rrnD-rrnE)1 rph-1*	CGSC
YM63	BW25113 P_*lacZ*_ *-topA*76(ts) *zci-2234*::*cat*	this study
YM64	BW25113 P_*lacZ*_ *-topA*76(ts) *zci-2234*::*cat ΔtopB761*::*kan*	this study
T4+	T4D	[16]
T4 K10	*38amB262 51amS29 nd28* (*denA*) *rIIPT8* (*ΔdenB-rII*)	[37]
T4 K10 *55*.*2*	K10 *55*.*2* (ATG-> ACA)	this study
T4 *39*	T4D *39amEA29*	R.H. Epstein’s collection
T4 *55*.*2*	T4D *55*.*2* (ATG-> ACA)	this study
T4 39 55.2	T4D *39amEA29 55*.*2* (ATG-> ACA)	this study

^a^All strains are *E*. *coli* K-12 derivatives except B^E^ and BL21(DE3), which are *E*. *coli* B strains.

### Plasmid based lethality assay

Plasmid based lethality assays were performed as previously described [31]. Briefly, bacteria were cultivated overnight in LB with Kn and Ap to maintain both the *55*.*2* and the *topA* plasmids. The next morning, saturated cultures were diluted 1/80 in M63 medium supplemented with 0.2% glucose and Kn but without Ap and grown to *A*_600 nm_ = 0.4, before spreading dilutions on M63 agar plates supplemented with 0.2% glucose, Kn, 600 μM IPTG, and 80 μg ml^-1^ X-Gal. Colonies were counted and photographed after 36 h at 37°C.

### Purification of gp55.2-His_6_

The protein was expressed from pMCN1 in BL21(DE3) cells. Exponentially growing bacterial cultures were induced with 0.2% arabinose for 2 hours at 37°C. The protein was purified from frozen cell pellets on Ni-NTA agarose (#30210, Quiagen) according to manufacturer instructions. Eluted fractions containing the His–tagged protein were desalted on a PD-10 column (GE Healthcare) equilibrated in TKDG buffer (50mM Tris pH 7.4, 100mM KCl, 1mM DTT, 10% glycerol) and concentrated on a centricon microconcentrator (3 kDa molecular weight cut-off, #4202, Amicon). Protein concentration was determined by Bradford assay (Bio-Rad Protein Assay) and purity (>99%) was assessed by Coomassie brilliant blue staining of proteins separated by SDS-polyacrylamide gel electrophoresis.

### Electrophoretic Mobility Shift Assay (EMSA)

EMSA were performed with form I and form I’ DNA of plasmid pDB29 prepared as described below; linear DNA was obtained by restriction digestion with EcoRV. Plasmid DNA (0.3 μg, 79 fmol) were incubated with the indicated amounts of gp55.2-His_6_ in EMSA Buffer (50 mM Tris, 100 mM KCl, 1 mM β-mercaptoethanol, 1 mg ml^-1^ Bovine serum albumin (BSA), 10% glycerol, pH 7.5 at 25°C) for 30 min at 37°C. Samples were then electrophoresed at 3V cm^-1^ through 0.8% agarose gels in 1x TBE (90 mM Tris, 90 mM boric acid, 2.5 mM EDTA) for 20 h at 4°C with constant buffer recirculation. Gels were stained with ethidium bromide and photographed under UV light using an E-BOX VX5 system (Vilber Lourmat, France) or a Chemi Doc MP system (Bio-Rad Laboratories)

### DNA relaxation and supercoiling assays

Covalently closed, negatively supercoiled circular DNA (form I) of plasmid pDB29 [38] was obtained by standard alkaline lysis procedure, followed by cesium chloride density gradient centrifugation [39]. Covalently closed, relaxed circular DNA (form I’) was prepared by incubating form I DNA with wheat germ topoisomerase I (Promega, #M285) according to manufacturer instruction. All plasmid DNAs were extracted with phenol/CHCl_3_ and ethanol precipitated before being used as substrates in supercoiling reactions. For relaxation assays, *E*. *coli* Topo I was purchased from New England Biolabs (#M0301) and reactions were performed in 1x CutSmart buffer with 0.6 μg of form I pDB29 DNA (158 fmol). The indicated amounts of gp55.2-His_6_ were first incubated with DNA for 15 min at 37°C, then the indicated amounts of Topo I were added and samples were incubated at 37°C for 15 min. Reactions were terminated as described [40]. Briefly, EDTA was added to 25mM and samples were incubated for 2 min at 37°C; then, SDS and proteinase K were added to 1% and 100 μg/ml, respectively, and the samples were incubated for an additional 15 min at 37°C. DNA products were extracted with phenol/CHCl_3_ and electrophoresed at 2V cm^-1^ through 0.8% agarose gels in 1x TBE buffer for 48 h at 4°C with constant buffer recirculation. Gels were stained and photographed as explained above. For supercoiling assays, *E*. *coli* DNA gyrase was purchased from New England Biolabs (#M0306) and reactions were performed in 1x Gyrase buffer with 0.6 μg of form I’ pDB29 (158 fmol). The indicated amounts of gp55.2-His_6_ were first incubated with DNA for 15 min at 37°C, then the indicated amounts of DNA gyrase were added and reactions were further incubated at 37°C for 30 min. Reactions were terminated and analyzed as described for the relaxation assays.

### *In vivo* analysis of plasmid topoisomers

To obtain monomeric molecules, plasmids were linearized and self-circularized in large ligation volumes. The ligations were transformed into the appropriate strains and independent clones were purified and used for subsequent analyses. Equal amounts (A_600nm_) of exponentially growing bacterial cultures were quickly chilled by mixing them with equal volumes of ice-cold growth medium and transferred on ice. DNA was extracted by standard alkaline lysis procedure, followed by phenol/CHCl_3_ extraction and ethanol precipitation. The nucleic acid pellets were dissolved in TE buffer with 2 μg ml^-1^ RNAse A and analyzed on 0.8% agarose/ TBE gels with 1 or 1.5 μg ml^-1^ chloroquine (CLQ) as described in the previous section. Loaded volumes were adjusted to account for the difference in plasmid copy number between *55*.*2*-expressing and control strains. After electrophoresis, gels were washed thrice for 15 min with 0.5x TBE, stained with ethidium bromide (1 μg ml^-1^) in H_2_O for 1 hour, destained 20 min with 1mM MgSO_4_, and photographed as described above. The migration of the same plasmid DNA samples on gels with two different CLQ concentrations allowed us to determine which bands correspond to more negatively supercoiled plasmids [41]. Densitometric analyses were performed on unsaturated images using the MultiGauge (v.3) software (Fujifilm LifeScience). The data were normalized to total amount of DNA in each lane. Two-dimensional gel (2D) gel analyses were performed as previously described [42] using 0.8% agarose/ TBE gels with 1.5 μg ml^-1^ and 25 μg ml^-1^ CLQ in the first and second dimension, respectively.

### Plasmid copy number quantification

Plasmid DNA was extracted from equal amounts of bacteria (A_600nm_) by standard alkaline lysis procedure and resuspended in TE buffer. Equal volumes were linearized with an appropriate restriction enzyme, ran on standard agarose TAE gels, and visualized with ethidium bromide. Unsaturated images were analyzed using the MultiGauge (v.3) software (Fujifilm LifeScience) and signals were calibrated using a standard curve of diluted High Mass DNA ladder (Invitrogen) loaded on each gel.

### Intracellular phage growth and phage competition assays

Intracellular phage growth assays were performed as previously described [29] with the following modifications. CR63 bacteria were grown in M9 medium supplemented with 50 μg ml^-1^ tryptophan and 0.4% glucose without (M9) or with 1% casmino acids (M9S) to a concentration of 1 x 10^8^ cfu ml^-1^ and placed on ice. Before infection, bacteria were prewarmed for 10 min and infection (t = 0) was started by mixing bacteria with an equal volume of phages diluted in the corresponding growth media. Phage growth was carried at the indicated temperature with vigorous agitation. Competition assay were performed with CR63 cells grown in M9S. For each growth cycle, an aliquot of bacteria was prewarmed 10 min at 37°C, mixed with an equal volume of phage diluted in M9S (1 x 10^6^ pfu ml^-1^, multiplicity of infection (moi) = 0.01), and growth was carried at 37°C with vigorous agitation. After 45 min, the cultures were lyzed with a drop of CHCl_3_ placed one ice for 10 min, transferred to another tube, and kept at 4°C. Because the average burst size under these conditions was 130, the lysates were diluted 65 times in M9S and directly used to carry the next growth cycle. The titer of each lysate was determined to ensure that the moi of the next growth cycle never exceeded 0.1.

### Determination of the ratio of *55*.*2* ^+^ to total phage

Aliquots from each phage lysates (≈1 x 10^5^ pfu) were used as a template for amplification with primers 55.3up (5’-ggaaatttaaatgaatcctgaatc) and 55.1dn (5’-agacctatcttaaagcatagag) using the Taq DNA polymerase (Invitrogen) following manufacturer’s instruction. Aliquots of the PCR were digested with BsrGI, which cuts only at the mutated *55*.*2* initiation codon in the amplified fragment. Restriction digests were run on 1% agarose/TAE gels, stained with ethidium bromide, imaged under UV light, and the intensity values of the DNA bands were determined on unsaturated images using the Multi Gauge 3.0 software (FujiFilm). The K10/ (K10-*55*.*2* + K10) ratios were calculated by dividing the intensity value of the undigested band by the cumulated intensity values of the undigested band and the two bands resulting from BsrGI digestion. We confirmed the accuracy of the PCR-restriction determination by testing a standard curve prepared with known amounts of K10 and K10-*55*.*2* phage. In addition, the ratio of selected growth cycle lysates was confirmed by the determination of the genotype of individual plaques (n = 20).

## Results

### Ectopic expression of cloned phage T4 gene *55*.*2* inhibits irreversibly *E*. *coli* growth

**Fig 1:**
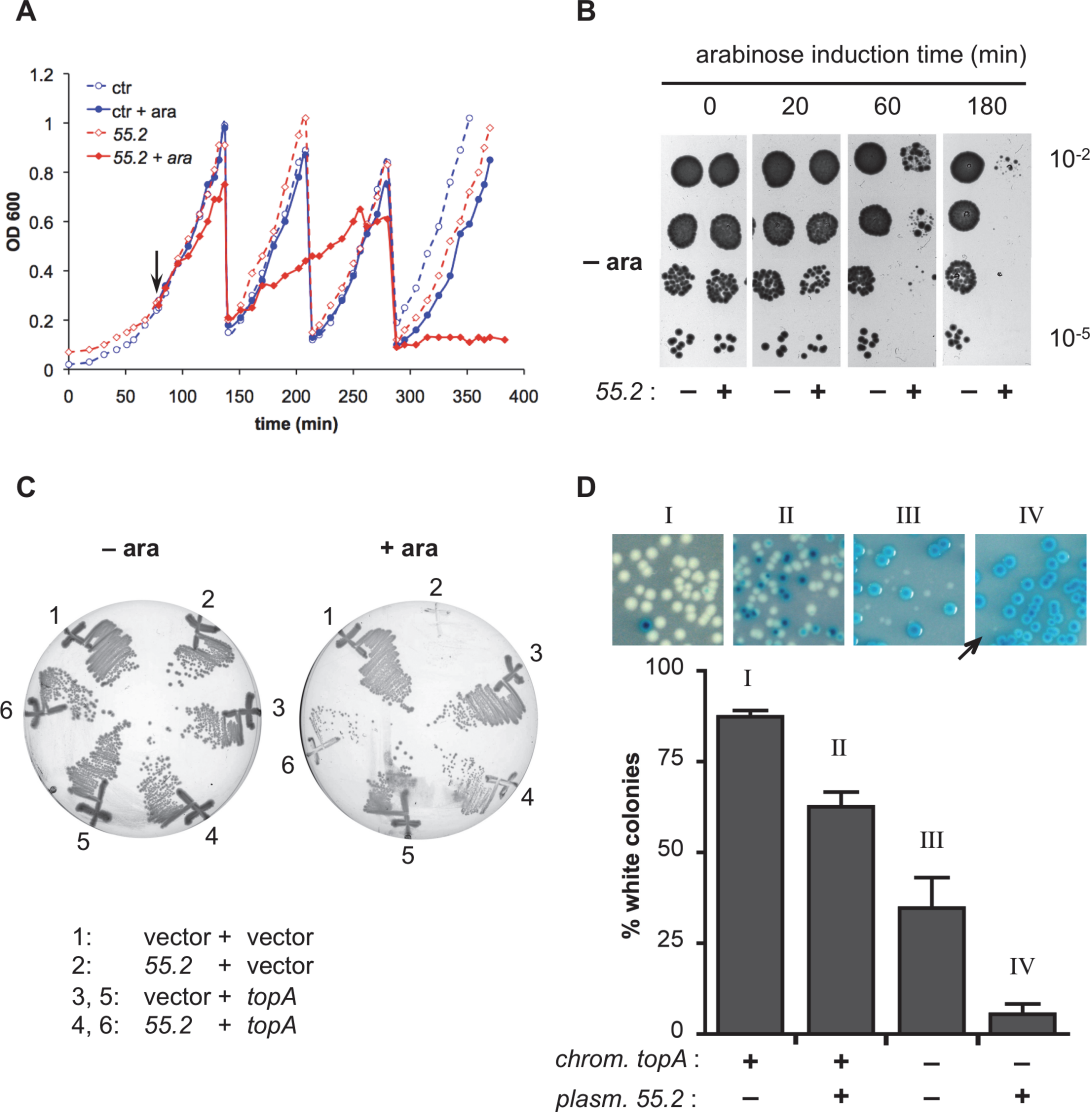
The toxicity of *55*.*2* in *E*. *coli* is suppressed by an increase in the copy number of *topA*. (A) Liquid growth assay. Overnight cultures of DB503 cells harboring pBAD101 (ctr) or pDB2114-101 (*55*.*2*) plasmids were diluted in fresh LB medium and optical density (A_600nm_) was measured at the indicated times. At A_600nm_ = 0.3, cultures were split in two and one half was induced with 0.2% arabinose (vertical arrow). When A_600nm_ reached ≈ 1, cultures were diluted 10-fold in prewarmed medium plus or minus arabinose. The graph depicts the data of a representative experiment. (B) Reversibility assay. During a growth curve assay, aliquots of arabinose-induced DB503 cultures, harboring pBAD101 (*55*.*2* –) or pDB2114-101 (*55*.*2* +) plasmids, were withdrawn at the indicated times, washed in cold media without arabinose, and adjusted to the same A_600nm_. Serial 10-fold dilutions were spotted on LB plates without arabinose. (C) DB503 cells transformed with pBAD101 (vector) or pDB2114-101 (*55*.*2*) and one of the compatible plasmids, pDB868-2 (vector, 1–2), pDB34-8-4 (*topA*, 3–4), or pDB34-8 (*topA*, 5–6) were streaked on LB plates with or without 0.2% arabinose. (D) Plasmid based lethality assay. Overnight cultures of AS1047 (*topA* +) or AS1050 (*topA*-) transformed with pBAD33-K (*55*.*2* –) or pDB2114-33-K (*55*.*2* +) were diluted and outgrown as indicated in the Materials and Methods section. Aliquots were diluted, and plated on M63 plates supplemented with glucose and X-gal. The number of blue and white colonies was scored after 36h at 37°C. Representative photographs are shown in the upper panel (the position of a rare white colony in inset IV is indicated by an arrow). The lower panel depicts percentage of white colonies; average and standard deviation are from three independent experiments.

Using vectors expressing T4 genes under the control of an inducible arabinose P_BAD_ promoter, we previously demonstrated that the ectopic expression of the *55*.*2* ORFan was deleterious for *E*. *coli* growth [29]. Such plasmids confer an arabinose sensitive phenotype (Ara^S^) on the carrier host strain. In these experiments, performed with a medium copy number plasmid derived from ColEI, the residual expression of *55*.*2* from the P_BAD_ promoter in the absence of arabinose induction was sufficient to affect bacterial growth. Initial growth after dilution of the stationary phase cultures was delayed compared to that of bacteria harboring an empty vector control (S1A Fig). Furthermore, the colony forming units (cfu) in saturated cultures were 10-fold lower than in the controls (S1B Fig). To minimize the effects of residual expression from the repressed P_BAD_ promoter, the *55*.*2* ORFan sequence was cloned into pBAD101, a low-copy inducible vector with a pSC101 derived replicon [43]. This essentially abolished the phenotypes observed with the original construct in the absence of arabinose. However, in arabinose-induced conditions, *55*.*2* expression still prevented bacterial colony formation. Growth curve assays showed that the addition of arabinose led to a slow growth arrest that was complete only by 3 h (Fig 1A). However, a reversibility assay demonstrated that even after only one hour of *55*.*2* induction, there was a ~100 fold decrease in the capacity of induced cells to form colonies in the absence of arabinose (Fig 1B). Thus *55*.*2* has an irreversible bactericidal action.

### Overexpression of *topA*, the gene encoding the *E*. *coli* topoisomerase I, suppresses 55.2 toxicity

We searched for *E*. *coli* genes that, when present in multiple copies, suppressed the Ara^S^ phenotype induced by *55*.*2* expression (multicopy suppressors). Initial attempts using standard *E*. *coli* genomic libraries identified only non-specific suppressors that interfere with P_BAD_ induction. These non-specific suppressors included genes of the *araBAD* operon, which encodes enzymes that degrade arabinose, and *ydeA*, which encodes a transporter that facilitates arabinose export [44]. Thus, we prepared a new library from a strain that harbors both *araBAD* and *ydeA* deletions. The plasmid library was introduced by electroporation into cells carrying a pBAD101 plasmid with a *55*.*2* insert and transformants were directly selected on LB plates containing 0.2% arabinose. Plasmids from such suppressor strains were purified and the suppressor activity of the library plasmids verified. Non-specific suppressors were identified and eliminated as previously described [29]. After testing the equivalent of ≈ 180 *E*. *coli* genomes, we isolated one plasmid that conferred specific, albeit partial, resistance to *55*.*2* expression. The 4259 bp insert of this plasmid contained *yciN*, *topA* and part of *cysB*. Subsequent subcloning revealed that a region containing the entire *topA* coding sequence as well as 272 bp sequence upstream of the initiation codon sufficed for suppression (Fig 1C, streak 4). The product of *topA* is the DNA topoisomerase I (Topo I), a type IA topoisomerase that removes excess negative supercoils introduced by DNA gyrase into the *E*. *coli* chromosome and plasmids [45,46].

### Incompatibility of low-level expression of *55*.*2* with reduced expression of Topo I and Topo III

Since high-level expression of *topA* partially suppressed the *55*.*2*-induced toxicity, we examined the effect on *E*. *coli* of varying levels of expression of both *55*.*2* and *topA*. The viability of *topA* null mutants in the absence of compensatory DNA gyrase mutations has long been debated [47,48]. Recently, it was shown by Rudolph and coworkers that the loss of *topA* results in a severe growth defect [31]. In their assay, a copy of the *topA* gene cloned into an unstable mini-F *lac* ^+^ plasmid complemented a chromosomal *topA* deletion. In a *lac* ^—^strain, loss of the plasmid could be followed by colony color on X-gal plates. Using this system, we determined the effect of low-level expression of *55*.*2* on *topA* loss. *TopA* ^+^ and *ΔtopA* bacteria harboring the *topA* lac^+^ mini-F plasmid were transformed with a pBAD vector with or without a *55*.*2* insert. After outgrowth in the absence of antibiotic selection for the *topA* mini-F plasmid, bacteria were plated on minimal X-gal glucose plates with selection for the pBAD plasmid (Fig 1D). In the presence of a *topA* ^+^ chromosomal copy (I), most colonies were white indicating that the loss of the *topA* ^+^ mini-F plasmid had no effect on growth. In the absence of a *topA* chromosomal copy (III), about 65% of the colonies retained the *topA* ^+^ mini-F plasmid; importantly, the white colonies were notably smaller than the blue. These results confirm that *topA* mutants have a severe growth defect. When a *55*.*2*-coding plasmid was present, very few white colonies were observed (5.5%) and their size was significantly smaller than the white colonies obtained in the absence of a *55*.*2*-coding plasmid (compare insets IV to III). Thus, the *topA* function is apparently necessary to tolerate a low-level expression of *55*.*2* which is non-toxic in wild type cells. These results were obtained using a *55*.*2* expressing plasmid with ≈30 copies per cell and in the presence of glucose, which represses expression from the P_BAD_ promoter [49]. Consequently, the level of *55*.*2* expression that manifests incompatibility in a *topA* deletion strain is very low. Significantly, the presence of a *55*.*2*-coding plasmid also reduced the frequency of *topA* ^+^ mini-F plasmid loss also in a *topA* ^+^ genetic background (II). This is consistent with very low levels of *55*.*2* expression having some effect on the growth of wild-type bacteria (S1A and S1B Fig) and suggests that a mere duplication of the *topA* locus suffices to partially overcome this effect.

As mentioned in the introduction, *E*. *coli* possesses another type IA topoisomerase, Topo III (*topB*). We asked whether, in the context of low-level expression of *55*.*2*, Topo III could partially compensate a decrease in Topo I activity. A medium copy pBAD plasmid expressing *55*.*2* was transformed into *E*. *coli* strains harboring a temperature sensitive (ts) allele of *topA* driven by a lacZ promoter and either a wild-type or a disrupted *topB* gene. We then assessed the viability of these strains in the presence of various concentration of IPTG at 37°C, a temperature at which the Topo I produced by the *topA* ^ts^ allele is less active than the wild-type enzyme [50]. The results, presented in S1C Fig, confirm the incompatibility of reduced Topo I activity with the residual expression of *55*.*2* from the P_BAD_ promoter (upper panel). The incapacity of P_lacZ_-*topA ΔtopB* bacteria to grow in the absence of IPTG repeated the known non-viability of *topA topB* double mutants [48,51]. Importantly, the reduced viability phenotype caused by low-level expression of *55*.*2* was stronger in the absence of a functional Topo III (lower panel). This further decrease in viability caused by *55*.*2* in the *ΔtopB* strain was already evident in the presence of 1mM IPTG, a concentration at which the *topB* ^+^ and *ΔtopB* strains harboring the control vector show no difference in viabilty. These results indicate that *E*. *coli* requires a minimal type IA topoisomerase activity to tolerate low-level expression of *55*.*2*.

### Inhibiting the GyrB ATPase subunit of gyrase reduces *55*.*2*-induced toxicity

**Fig 2:**
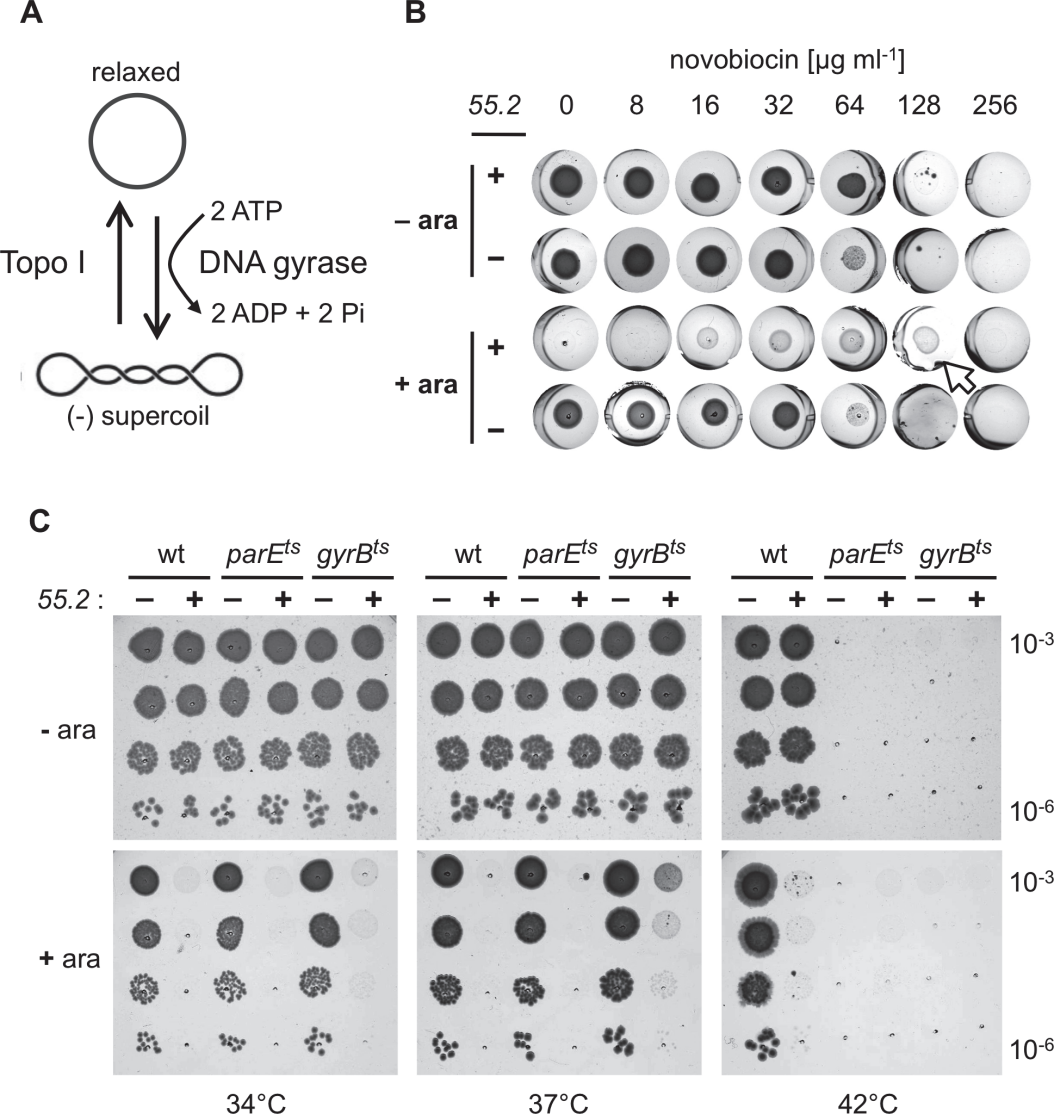
Partial inhibition of DNA gyrase ATPase activity protects from *55*.*2*-induced toxicity. (A) Outline of the regulation of the steady-state level of DNA supercoiling by the opposite actions of DNA gyrase and Topo I. (B) Novobiocin sensitivity assay. Overnight cultures of DB503 bacteria transformed with pBAD101 (*55*.*2 –*) or pDB2114-101 (*55*.*2 +*) were spotted on 24 wells LB agar plates with and without 0.2% arabinose containing the indicated amounts of novobiocin, and incubated overnight at 37°C. (C) W3110 (wt), CL001 (*parE* ^ts^), and BP199 (*gyrB* ^ts^) were transformed with pBAD101 (*55*.*2 –*) or pDB2114-101 (*55*.*2 +*). Dilution of overnight cultures were spotted on LB agar plates with and without 0.2% arabinose and incubated overnight at the indicated temperatures.

The supercoiling level of the *E*. *coli* chromosome and its plasmids is primarily determined by the opposing activities of Topo I, which removes negative supercoils and DNA gyrase (*gyrAB*), which adds negative supercoils (Fig 2A). Thus, inhibition of DNA gyrase would be expected to have a similar effect as increasing Topo I activity. Consequently, it seemed likely that such an inhibition would counteract the *55*.*2*-induced toxicity. We tested the sensitivity of *55*.*2*-expressing *E*. *coli* cells to novobiocin, an aminocoumarin type antibiotic that inhibits gyrase’s DNA supercoiling by competing with ATP for binding to the GyrB subunit [52–54]. As before, arabinose inhibited the growth of bacteria harboring a *55*.*2* coding pBAD101 plasmid. However, low concentrations of novobiocin (between 16 and 64 μg ml^-1^), which had no effect on the growth of bacteria carrying the control plasmid, partially reduced the toxicity associated with *55*.*2* expression (Fig 2B). Furthermore, *55*.*2* expression permitted bacterial growth of bacteria at 128 μg ml^-1^ of novobiocin, a concentration that inhibited growth in the absence of arabinose or when they harbored an empty pBAD101 plasmid (see arrowhead in Fig 2B).

This suggested that limiting DNA gyrase function alleviated, at least partially, the growth inhibition mediated by *55*.*2*. However, novobiocin can also inhibit the topoisomerase IV (Topo IV) of *E*. *coli* by binding to its ParE subunit, which is homologous to GyrB [55]. Thus, we asked whether partial inactivation of either DNA gyrase or of Topo IV sufficed to suppress *55*.*2* toxicity. We transformed *gyrB* and *parE* ts mutant strains with a *55*.*2* coding plasmid, and tested their sensitivity to arabinose at different temperatures. While *55*.*2* expression blocked bacterial growth in all strains at the permissive temperature (34°C), an intermediate temperature (37°C), which permitted the growth of the bacteria carrying either one of the two ts alleles, led to partial suppression of *55*.*2*-induced toxicity in the *gyrB* ^ts^ but not in the *parE* ^ts^ mutant strain (Fig 2C). Complete inhibition of DNA gyrase at 42°C abolished growth independently of *55*.*2* expression. Thus, we conclude that partial inhibition of GyrB is sufficient to offset the toxicity due to high-level expression of *55*.*2*.

### Gp55.2 binds to DNA *in vitro*

**Fig 3:**
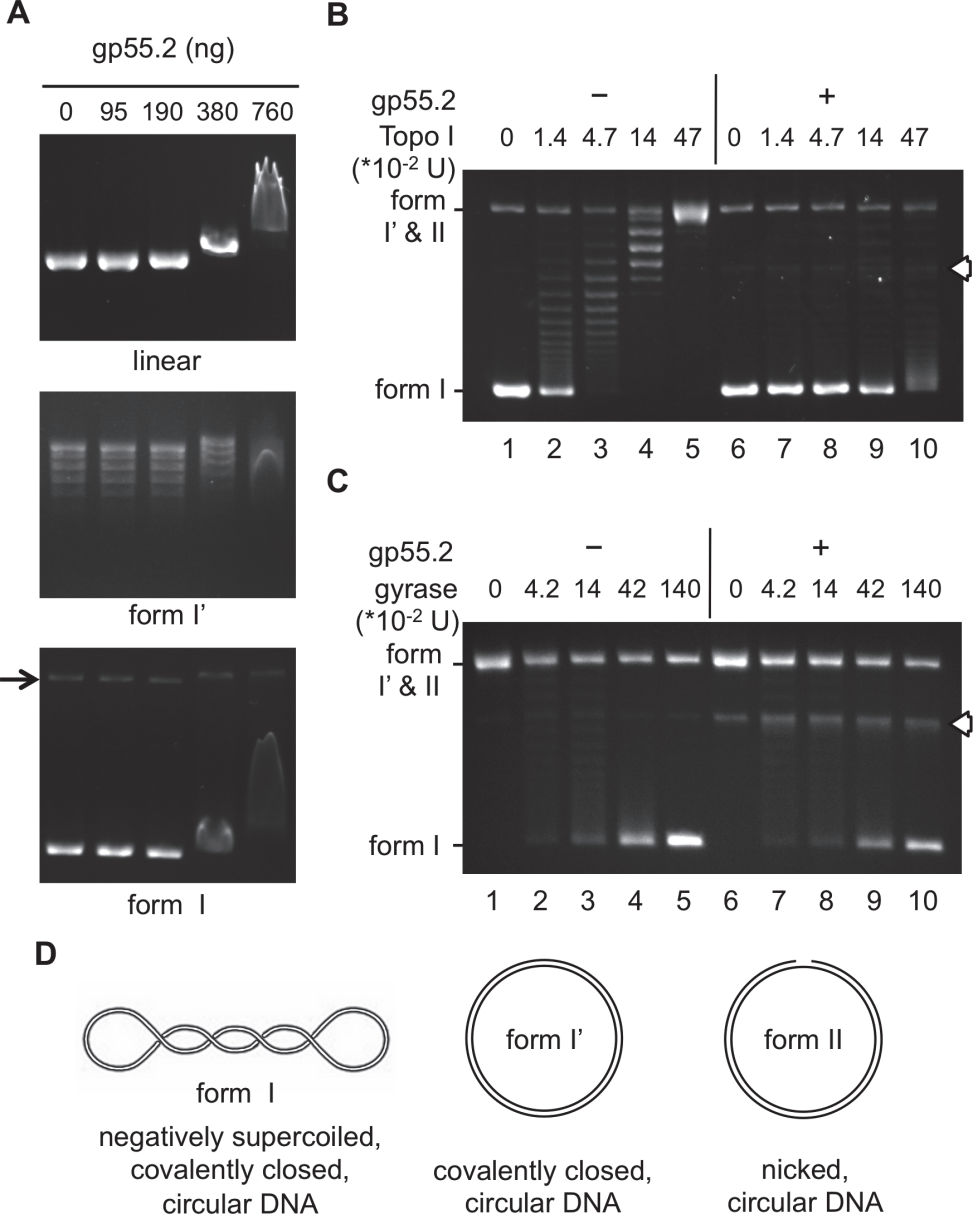
Gp55.2 binds to DNA, inhibits Topo I relaxation activity but does not affect DNA gyrase supercoiling activity. (A) Electrophoretic mobility shift assays (EMSA). Mixtures containing 300 ng (= 79 fmol) of linear, form I’, or form I pDB29 DNA and the indicated amount of gp55.2-His_6_ (95 ng = 6.9 pmol, namely one gp55.2 molecule per 65.6 bp for the amount DNA used in this assay) were incubated and analyzed as described in Materials and Methods. An arrow indicates the migration position of the form II DNA contaminating the form I DNA. (B) Relaxation assays mixtures containing 600 ng (= 158 fmol) form I pDB29 DNA and the indicated units of Topo I (0.47 U = 415 fmol) were incubated in the presence (+) or absence (–) of 855 ng of gp55.2-His_6_ (= 63 pmol, one gp55.2 molecule per 14.5 bp of DNA), and the DNA products were analyzed as described in Materials and Methods. The migration positions of form I, form I’ and form II DNA are indicated on the left; linear DNA migration position is indicated by an arrowhead. Results representative of two independent experiments are shown. (C) Supercoiling assays mixtures containing 158 fmol form I’ pDB29 DNA and the indicated units of DNA gyrase (0.42 U = 196 fmol) were incubated in the presence (+) or absence (–) of 855 ng of gp55.2-His_6_ and the DNA products were analyzed as described in the Materials and Methods section. Results representative of two independent experiments are shown. (D) Representation of form I, form I’ and form II plasmid DNA. Note that the treatment of form I plasmid DNA by a eukaryotic Topo I to obtain form I’ DNA results, at equilibrium, in a distribution of relaxed DNA topoisomers (A, middle panel) whose maximum corresponds to the fully relaxed form I’ plasmid illustrated in D.

Next, we asked whether gp55.2 could bind to DNA. The *in vitro* binding of gp55.2 to DNA was demonstrated by electrophoretic mobility shift assays (EMSA) employing purified gp552-His_6_ protein and plasmid DNA from pDB29 (a pBR322 derivative containing a mouse urokinase cDNA insert; [38]). As shown in Fig 3A, gp55.2 caused linear pDB29 DNA (upper panel) to migrate more slowly. This shift was easily detectable at a ratio of one gp55.2 molecule per 16.4 bp of DNA (380 ng of gp55.2 to 300 ng of DNA). At this protein to DNA ratio, gp55.2 also retarded the migration of covalently closed, relaxed circular DNA (form I’, middle panel), of covalently closed, negatively supercoiled circular DNA (form I, lower panel), and of the small amount of nicked circular DNA (form II, lower panel, arrow) that contaminated the form I DNA. Additional EMSA using M13 phage DNA showed that gp55.2 retarded the migration of both the double-stranded replicative form and the circular single-stranded virion DNA (S2A Fig). Thus, gp55.2 appears to bind negatively supercoiled, relaxed, and single-stranded DNA.

### *In vitro* gp55.2 inhibits Topo I but does not stimulate gyrase activity

The genetic evidence combined with the capacity of gp55.2 to bind to DNA suggests that this protein could either inhibit Topo I activity or stimulate that of DNA gyrase. We have directly examined the *in vitro* effect of gp55.2 on *E*. *coli* Topo I and DNA gyrase activities. The relaxing activity of Topo I was measured in the presence or absence of purified gp552-His_6_ at a concentration slightly higher than the lowest concentration at which a band shift was observed in the EMSA assays. As shown in Fig 3B, gp55.2 strongly inhibited the relaxation activity of Topo I, even at the lowest molar ratio of gp55.2 to Topo I tested (150:1; lane 10). Based on the amount of enzyme required to obtain similar relaxation levels in the absence and presence of gp55.2, we estimated that the relaxation activity was inhibited by 10 to 30 fold (compare lanes 2 and 3 to lane 10). Further experiments, in which the amount of Topo I was fixed and that of gp55.2 varied, indicated that a ratio of one gp55.2 molecule per 7 bp of DNA was required for complete inhibition (S3A Fig, lane 8). However, at lower gp55.2 to DNA ratio, both more supercoiled and more relaxed topoisomers were observed in the presence than in the absence of gp55.2 (S3A Fig, lane 4 to 7), suggesting that gp55.2 could also stimulate Topo I activity at low protein to DNA ratio. In stark contrast, gp55.2 had no effect on the negative supercoiling activity of DNA gyrase (Fig 3C). We also tested whether gp55.2 could inhibit the relaxation of supercoiled DNA by wheat germ Topo I, a type 1B topoisomerase whose structure and mechanism of relaxation are unrelated to these of *E*. *coli* Topo I [6]. The enzymatic activity of wheat germ Topo I was largely insensitive to gp55.2; furthermore, the observed limited inhibition of relaxation (≤ 20%) was independent of the concentration of gp55.2 used, suggesting that it reached saturation (S3B Fig). Altogether, these results show that gp55.2 inhibits specifically *E*. *coli* Topo I *in vitro*.

### *55*.*2* affects DNA topology control *in vivo*

**Fig 4:**
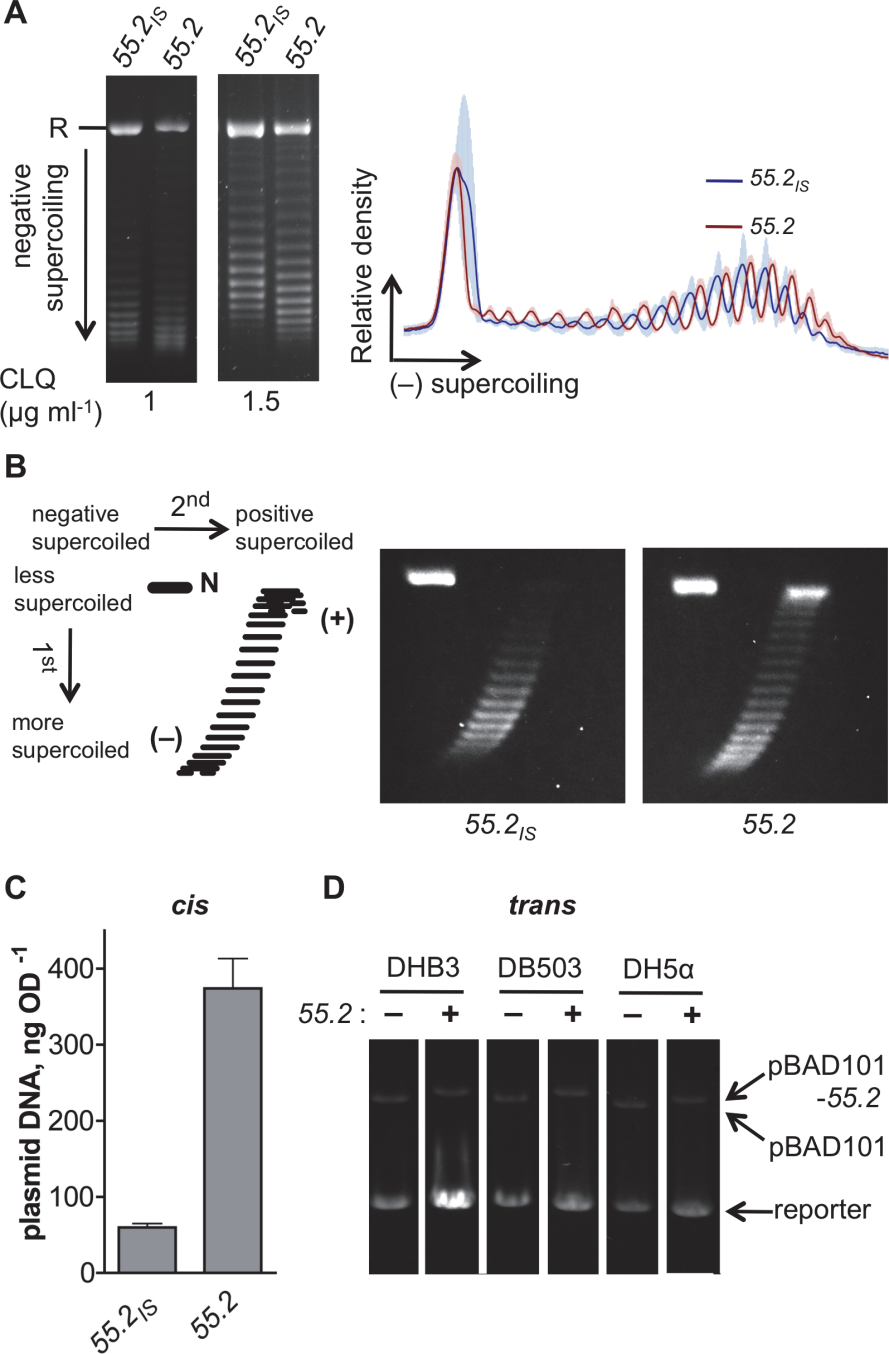
*55*.*2* expression affects the control of DNA topology and plasmid copy number in *E*. *coli*. (A) Plasmid topoisomers analysis. Left panel: plasmid DNA was extracted from exponentially growing DH5α harboring pYM58 (*55*.*2*_IS_) or pDB2114 (*55*.*2*) plasmids. Plasmid topoisomers were resolved on TBE agarose gels containing the indicated amount of CLQ. The position of migration of relaxed and/or nicked circular DNA is indicated (R). Right panel: Densitometry analysis of the topoisomer distribution on 1.5 μg ml^-1^ CLQ gel of four independent samples of pYM58 or pDB2114 plasmid DNA (gel images are depicted in S4A Fig). Plotted is the average (lines) and standard deviation (shaded area) of relative plasmid density as a function of negative supercoiling. (B) 2D electrophoretic separation of plasmid topoisomers. Left panel: schematic representation of a 2D gel. The migration positions of negatively supercoiled (–), positively supercoiled (+), and form II topoisomers (N) are indicated. Right panel: gel images show the 2D topoisomer distribution of plasmid DNA samples prepared as in A. Chloroquine concentration was 1.5 μg ml^-1^ and 25 μg ml^-1^ in the first and second dimension, respectively. (C) Plasmid copy number analysis. Linearized plasmid DNA samples from the experiment shown in A were quantified and normalized to the amount (A_600nm_) of bacteria used to extract the plasmids. The data represents means and standard error of four independent cultures. (D) Plasmid DNA was extracted from overnight cultures of DHB3, DB503, and DH5α transformed with pBAD101 (*55*.*2* –) or pDB2114-101 (*55*.*2* +), and a reporter plasmid (pDB868-2). Linearized plasmids were analyzed by agarose gel electrophoresis.

We investigated the effect *in vivo* of low, non-toxic levels of *55*.*2* expression on the supercoiling of plasmid DNA. To do this, we used pDB2114, a pBAD-*55*.*2* plasmid with an intermediate copy number, as a source of *55*.*2* expression, due to residual expression from P_BAD_ in the absence of arabinose. The same plasmid also served as the reporter of DNA supercoiling. As a control, we used a closely related plasmid in which the *55*.*2* coding sequence had been disrupted by a 5bp insertion after the ATG initiation codon producing a frame-shifted 18 amino acid peptide instead of gp55.2. This frame shift abolished all gp55.2 phenotypes of the parent plasmid in either the presence or absence of arabinose. Plasmid DNA was extracted from bacteria growing exponentially in the absence of arabinose and analyzed by one dimension agarose gel electrophoresis in the presence of chloroquine, an intercalating ligand that adds positive supercoils to plasmid DNA and allows the separation of supercoiled topoisomers [56]. As seen in Fig 4A, the distribution of topoisomers was slightly different between plasmids carrying the intact or the mutated copy of *55*.*2*. Changes in the distribution of slow migrating, partially relaxed topoisomers were more evident in the gel containing 1 μg ml^-1^ CLQ, while the fast migrating, negatively supercoiled topoisomers were better resolved in the gel containing 1.5 μg ml^-1^ CLQ. The densitometry profiles in the right panel summarize the analysis of four independent DNA samples for each plasmid (see also S4A Fig). Three principal differences can be observed. The slight shift between the two profiles is due to the 5 bp difference in the size of the two plasmids. It has been theoretically predicted, and experimentally demonstrated, that small changes in size (<10bp), which do not affect perceptibly the mobility of linear or form II (nicked circle) DNA, will cause a size-dependent decrease in the gel mobility of topoisomers with the same linking number. The half a turn shift we observed is perfectly in accordance with the published results [57]. Aside from this size-dependent change, two relevant differences were observed. First, the *55*.*2*-coding plasmid had a broader distribution of topoisomers compared to the control plasmid. In addition, the peak of the topoisomer distribution for the *55*.*2*-coding plasmid was shifted towards negative supercoiling by about half a helical turn. To obtain a better resolution of the relaxed topoisomers, the supercoiling state of each plasmid was also analyzed using two-dimensional chloroquine gels. The results presented in Fig 4B clearly show that low-level expression of *55*.*2* caused the appearance of both more relaxed and more negatively supercoiled topoisomers. The early stop codon in the *55*.*2* mutant plasmid used as a control could cause premature Rho-dependent transcription termination [58]. Because transcription elongation affects the *in vivo* supercoiling level of plasmids [59], we performed a control experiment to determine the potential effect of such transcriptional polarity. We compared two pBAD plasmids containing either the wild type *55*.*1* gene, a T4 ORFan adjacent but functionally unrelated to *55*.*2*, or a mutated *55*.*1* version in which the ATG start codon was changed to AGG. Both plasmid DNAs displayed almost identical topoisomer distributions; the only difference was a slight increase in the amount of the more negatively supercoiled topoisomers for the plasmid with the wild-type *55*.*1* sequence (S4B and S4C Fig). However, this increase was much less marked than in the case of the wild-type *55*.*2* plasmid. Thus, we conclude that low-level non-toxic expression of *55*.*2* has an effect on the regulation of plasmid DNA supercoiling in *E*. *coli*.

To determine if chromosomal DNA supercoiling was also affected by *55*.*2* expression, we measured the level of *topA* and *gyrA* mRNA since the transcription of both genes is determined, in part, by the supercoiling of their promoter regions. The transcription rate of *topA* increases with the increased negative supercoiling while that of *gyrA* increases with DNA relaxation [60–62]. Total RNA was extracted from bacteria harboring either a *55*.*2*-coding pBAD101 plasmid, or the empty vector, and cultures were growing exponentially in the absence of arabinose. RNAse protection assays were performed using *topA* and *gyrA* probes (S4D Fig), and the relative ratio of the *topA* over *gyrA*-protected fragments was determined by densitometric analysis. As shown in S4E Fig, the *topA* to *gyrA* ratio was slightly lower in bacteria harboring the *55*.*2* plasmid indicating that low-level expression of *55*.*2* did not increase supercoiling at this two promoters and might even have somewhat reduced it.

### Low non-toxic level of *55*.*2* increases the copy number of plasmids whose replication is controlled by antisense RNA

In analyzing the effects on DNA supercoiling mediated by gp*55*.*2*, we noticed that the amount of DNA from the gp*55*.*2* encoding plasmid was systematically higher than that present in various control plasmids. This pBAD vector has an origin of replication derived from ColEI, whose replication requires the formation of an R-loop between the plasmid origin and the complementary RNAII that serves as a primer for DNA replication [63]. The formation of R-loops is increased by negative supercoiling and inhibited by the action of RNAse H1 and Topo I [59]. To quantify the effect described above, aliquots of the plasmid DNA prepared for the supercoiling experiment described in Fig 4A were linearized by restriction digestion, quantified on agarose gel by comparison with a DNA standard, and normalized to the total amount of bacteria used to extract the plasmid. The results show that the amount of *55*.*2*-coding plasmid was seven times greater than that of a *55*.*2*-mutant plasmid (Fig 4C). Similarly, in overnight cultures of DH5α and DB503 (S4F Fig), the copy number of a *55*.*2*-coding plasmid was also increased five and two fold compared to a control plasmid. We then determined whether the presence of a gp55.2 coding sequence operated only in *cis* or whether gp55.2 also acted in *trans*. Bacteria were transformed with a *55*.*2*-coding pBAD101 plasmid and a reporter plasmid with a p15A-derived replication origin, which is related to the ColEI replicon [64]. Plasmid DNA was extracted from equal amounts of overnight cultures and linearized by restriction digestion. Compared to an empty control, the *55*.*2*-coding plasmid increased the quantity of the reporter plasmid in all three of the strains tested (Fig 4D). This demonstrates that even very low levels of gp55.2 expression (no arabinose was present in the cultures and the *55*.*2* gene was on a low copy plasmid) can affect plasmid copy number regulation in *trans*. Interestingly, *55*.*2* did not noticeably affect the copy number of pBAD101 (upper band in Fig 4D), whose replication is RepA dependent [65,66]. These results confirm the interesting and unexpected observation that gp55.2 significantly increases the copy number of plasmids whose replication is dependent on R-loop formation. This effect is entirely compatible with the observed increased plasmid supercoiling.

### A null mutation in gene *55*.*2* decreases the fitness of phage T4

**Fig 5:**
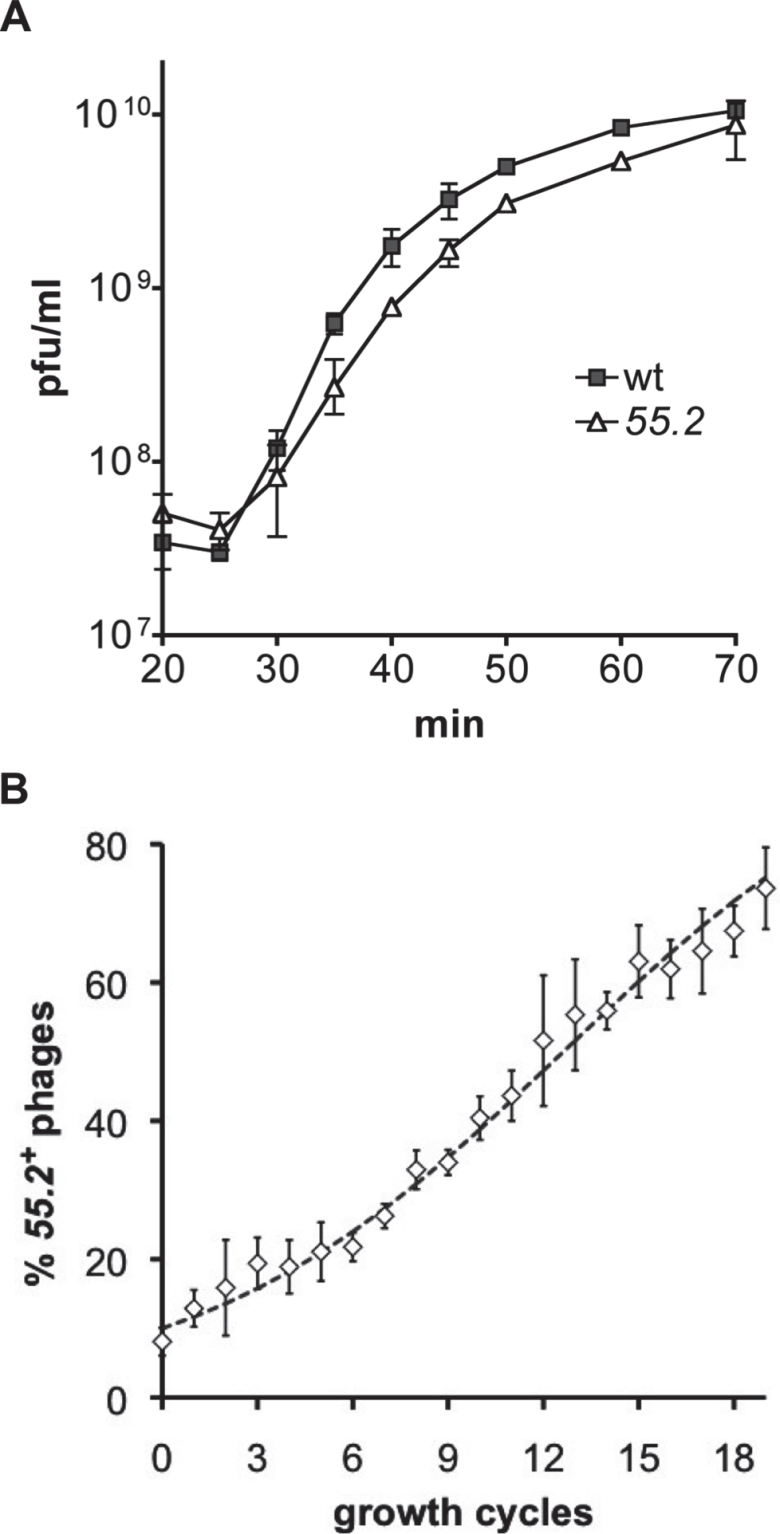
Loss of gene *55*.*2* function reduces T4 phage fitness. (A) CR63 cells grown in M9 medium were infected with T4 K10 (wt) or T4 K10-*55*.*2* (*55*.*2*) at a moi of 6 at 30°C. Intracellular phage accumulation was followed at the indicated time points; free phages at 25 min represented < 12% of the total infective centers. Data represents mean and ranges of two (wt) and four (*55*.*2*) independent experiments. (B) Competition experiment. A mix of T4 K10 and T4 K10-*55*.*2* with an initial ratio of 1:9 was grown on *E*. *coli* CR63 in M9S medium at 37°C at low moi (< 0.1) over successive growth cycles. The percentage of *55*.*2+* phages was determined by PCR and digestion as described in Materials and Methods. Data represent mean and standard deviation of four independent experiments; the dotted line represents the result of a simulation were the *55*.*2* mutant has a 16% disadvantage per growth cycle. The intracellular phage accumulation over a single growth cycle in these conditions is shown in S5A Fig.

Most T4 ORFans are probably “non-essential genes” since many lie in genomic regions that can be deleted without noticeably affecting phage yield [24]. However, it has not been excluded that the absence of gp55.2 function could influence the production of T4 progeny under standard laboratory conditions. We used the T4 I/S system [37] to replace the ATG initiation codon of *55*.*2* with an ACA (threonine) codon in the genome of the K10 T4 strain; an analogous substitution on a plasmid completely abolished the *55*.*2*-induced Ara^S^ phenotypes (data not shown). The plaque morphology of the resulting *55*.*2* mutant was indistinguishable from that of the K10 parent strain (data not shown). However, this *55*.*2* mutant phage exhibited a small but reproducible five minutes delay in the accumulation of intracellular viral particles in minimal media. Nonetheless, it eventually achieved a burst size similar to that of the parental strain (Fig 5A). A comparable delay was also observed in complete media at 37°C (S5A Fig). In order to determine whether such a small difference could change the overall fitness of the *55*.*2* mutant strain, we compared its growth with that of the parental strain over successive growth cycles. In these experiments, a mixture of control and mutant phages was grown on *E*. *coli* bacteria; the progeny of this initial growth cycle was used to perform a second growth cycle and so forth. Importantly, all infections were performed at a low moi (< 0.1) to minimize co-infection and complementation by the wild type phage. The initial phage mix contained 90% K10-*55*.*2* mutant and 10% K10 wild-type phages because a decreased fitness of the *55*.*2* mutant was anticipated. After 19 growth cycles, the *55*.*2* mutant phage represented less than 30% of the population (Fig 5B). A simulation showed that such results could be obtained if the mutant phage has a 16% growth disadvantage per growth cycle (dotted line in Fig 5B). Hence, we conclude that, although *55*.*2* is a “non-essential gene”, it nevertheless confers a non-trivial growth advantage to T4 even under standard laboratory conditions.

## Discussion

We have previously identified *55*.*2* as a T4 ORFan whose ectopic expression is toxic to uninfected *E*. *coli* [29]. When cloned in a medium copy pBAD plasmid (ColEI replicon), the low-level “leaky” expression of *55*.*2* from the P_BAD_ promoter in the absence of arabinose caused an increased lag phase in the growth of diluted liquid cultures and a decreased bacterial titer at saturation. These effects were not detected with a lower copy plasmid (pSC101 replicon) in the absence of arabinose. Induced expression of gp55.2 in the presence of arabinose led to irreversible growth arrest in both plasmid backgrounds.

Further analysis identified two ways that partially suppress this *55*.*2*-mediated bacterial killing: multicopy expression of Topo I or the partial inhibition of GyrB activity. These results suggested that gp55.2 was acting by either inhibiting Topo I activity or by increasing DNA gyrase activity. A plasmid-based lethality assay clearly demonstrated that a low-level, normally non-toxic, expression of gp55.2 is not compatible with reduced Topo I activity. This incompatibility was independently confirmed using a strain whose *topA* expression could be regulated by IPTG. Interestingly, in the absence of Topo III, the second type IA topoisomerase of *E*. *coli*, bacteria expressing low levels of gp55.2 required higher expression of Topo I to grow. Taken together, these results support the notion that *55*.*2* inhibits DNA relaxation and/or stimulates DNA supercoiling.

*In vitro* experiments demonstrated that gp55.2 inhibits the relaxing activity of *E*. *coli* Topo I but that it does not affect the introduction of negative supercoils by *E*. *coli* gyrase. The inhibition of Topo I activity by gp55.2 could result from a direct protein-protein interaction. Alternatively, gp55.2 binding to DNA could alter the double helix conformation and/or occlude the sites at which Topo I binds. Increased Topo I expression has been shown to suppress the toxicity associated with Tn*5* transposase (Tnp) overproduction [67,68]. A direct interaction between the two proteins is responsible both for the *in vitro* inhibition of Topo I activity and for the *in vivo* suppression of Tnp toxicity. We failed to detect an interaction between gp55.2 and Topo I using *in vivo* pull-down assays (data not shown). Although we might not have found the optimal condition to detect such an interaction, there are nonetheless other arguments that disfavor this hypothesis. Firstly, an alteration of DNA topology is not required for the suppression of Tnp toxicity and overexpression of a partially defective mutant of Topo I can suppress Tnp toxicity. Conversely, we showed that partial inhibition of gyrase partly suppressed gp55.2 lethality, even though it should reduce *topA* expression [60,62]. Second, the molar ratio of protein to Topo I required to observe *in vitro* inhibition is far lower for Tnp than for gp55.2 (1–10:1 vs. >150:1). The high concentration of gp55.2 required for Topo I inhibition supports the alternative site occlusion/DNA conformation hypothesis. Such a mechanism explains the inhibition of Topo I by the nucleoid-associated protein (NAP) HU at high protein to DNA ratio [69]. EMSA showed that gp55.2 and Topo I, at a 35:1 molar ratio, could bind simultaneously to plasmid DNA (S2B Fig). Nevertheless, occlusion could take place at the higher gp55.2 to Topo I ratio required to detect inhibition (≥ 150:1). We also tested whether gp55.2 could alter DNA topology on its own. The incubation of gp55.2 with relaxed DNA (form I’) in the presence of wheat germ Topo I showed that, under studied conditions, the phage protein could constrain neither negative nor positive supercoils (S3C Fig). Further studies will be needed to determine the precise mechanism of inhibition. One possibility could be that gp55.2 binds specific topological features favored by negative supercoiling, like the single-stranded DNA regions that are required for DNA supercoil relaxation by Topo I [70]. This hypothesis, inferred from the capacity of gp55.2 to bind single-stranded M13 DNA, is also supported by two observations. Firstly, gp55.2 only marginally affected the activity of wheat germ Topo I that does not require a single-stranded DNA substrate [6]. Secondly, at low protein to DNA ratio, gp55.2 seemed to both stimulate and inhibit Topo I activity. An ambivalent effect on Topo I activity was previously reported for the *E*. *coli* single-stranded DNA binding protein (SSB) that stimulates non-covalent interaction of Topo I with DNA by stabilizing single-stranded region at low protein to DNA ratio but that inhibits DNA relaxation at higher ratio [71].

The *in vivo* distribution of plasmid topoisomers indicates that low-level expression of *55*.*2* caused both the appearance of more negatively supercoiled and more relaxed topoisomers, resulting in a much broader distribution than in the absence of gp55.2. The measurement of the relative expression of *topA* and *gyrA* was compatible with unchanged or slightly reduced chromosome superhelical density in bacteria expressing gp55.2. These results appear counterintuitive since a complete defect of Topo I results in increased negative supercoiling of both plasmids and the bacterial chromosome [45]. However, the limited amount of gp55.2 produced from the uninduced P_BAD_ promoter is unlikely to completely inhibit Topo I activity. Actually, low-level *55*.*2* expression did not block the rapid relaxation induced by inhibition of DNA gyrase by norfloxacin (S4G Fig), a process which is almost entirely dependent on Topo I activity [72]. Furthermore, partial inhibition of Topo I should be compensated for by the homeostatic regulation of bacterial DNA supercoiling that takes place at both the enzyme activity and gene expression levels [73]. We also note that *in vitro* Topo I activity seemed to be both inhibited and stimulated by low gp55.2 to DNA ratio resulting in a broader topoisomer distribution than in the absence of gp55.2. The *in vivo* observations could be reconciled with the genetic and *in vitro* data if the inhibition of Topo I by gp55.2 were stronger on some DNA substrates than on others. DNA containing R-loops is a potential candidate. Indeed, low-level expression of 55.2 caused a strong increase in copy number of plasmids that use an R-loop for replication, and R-looped supercoiled plasmids are better substrate for Topo I than supercoiled plasmids without R-loops [74]. Topo I prevents excessive R-loop formation and *topA* null alleles are incompatible with deletions of *rnhA*, which encodes RNAse HI, an enzyme that degrades the RNA strand of R-loops [47,59,75]. Thus, the more severe phenotype induced by low levels of gp55.2 in the absence of RNAse H1 (S1C Fig) is consistent with a partial inhibition of Topo I and may reflect a preferential inhibition of Topo I activity on supercoiled DNA containing R-loops.

The *in vitro* experiments suggest that high levels of gp55.2 could completely inhibit Topo I *in vivo*. This is supported by the suppression of *55*.*2* lethality by *topA* overexpression or partial inactivation of DNA gyrase. Indeed, the thermosensitive mutation of *gyrB* (*gyrB203*) used in this study suppress the growth defect of a *topA* null mutation at 37°C [47]. Yet, Topo I inhibition cannot entirely explain gp55.2 killing. Indeed, there is now a consensus on the fact that Topo I is not absolutely required for growth [31,48]. Furthermore, both the plasmid-based lethality assay and the experiments with the tunable *topA* allele showed that low-level expression of 55.2 further diminished the growth capacity of bacteria devoid of Topo I. An interesting possibility could be that gp55.2 inhibits both Topo I and Topo III. As mentioned above, *topA topB* double mutants are non-viable. Although each topoisomerase has a different physiological role, Topo III is structurally and functionally related to Topo I and can relax negatively supercoiled DNA *in vitro* [76]. Indeed, we showed that bacteria required Topo III to tolerate low level of gp55.2 when Topo I activity was reduced.

Altogether, the simplest hypothesis to explain the data presented in this study is that gp55.2 inhibits both Type IA topoisomerases. Nevertheless, we cannot rule out that a reduction of DNA negative supercoiling is required to accommodate and suppress the consequence of a gp55.2 interaction with DNA, alone or in conjunction with a NAP. In this case, the *in vitro* inhibition of Topo I by gp55.2 would be an incidental consequence of gp55.2 binding to an intermediate DNA structure required for supercoil relaxation by Type IA topoisomerase.

Disruption of *55*.*2* in T4 phage showed that this gene is a *bona fide* non-essential gene, since the intracellular accumulation of virion particles was only slightly retarded in the null mutant. However, gp55.2 is conserved in many T4-related phages that infect different bacterial genera [25]. Competition assays showed that, even in standard laboratory conditions, *55*.*2* provided an easily detectable growth advantage. Thus, *55*.*2* must take part in one or several pathways required for optimal viral replication cycle. The toxicity of *55*.*2* as well as the characterization of its mRNA as an early transcript [77] suggests a role in the transition from host to phage metabolism or in phage DNA replication. The gp55.2 protein could play a role in the regulation of supercoiling by the phage. Although physically linear, the phage genome shows a transient acquisition of negative superhelicity that precedes T4 DNA synthesis and requires the host gyrase [78]. The function of DNA supercoiling during T4 infection is not clear: on the one hand, gyrase activity is not required for T4 DNA synthesis [22,79], but on the other hand, the burst size of T4 phage is reduced on a *gyrB* mutant host [22] and the release of superhelical tension slightly reduces the rate of T4 DNA synthesis [72]. Further investigation will be required to determine if the fitness provided by gp55.2 is directly linked to its modulation of host Topo I activity during infection. We have examined the importance of gp55.2 function in the absence of the phage topoisomerase. The intracellular growth curve of a *39am*, *55*.*2* double mutant was similar to the *39* single mutant, showing the extended eclipse period and reduced burst size characteristic of DNA delay mutants (S5B Fig). However, mature phage particle accumulation was slightly delayed in the double mutant, as observed in the comparison of the *55*.*2* single mutant with T4+. This additive effect suggests that gp55.2 and T4 topoisomerase act in independent biochemical pathways.

Despite T4 being one of the most studied phages, its genome codes for >100 ORFans whose biological functions, if any, are still largely unknown [24]. These genes are generally thought to be non-essential and they are largely regrouped in the so-called hyperplastic regions (HPR) of the T4 genome. Several hypotheses have been proposed to explain their maintenance through evolution. Some genes might contribute to the “molecular arms race” whereby resistance mechanisms and new ways to circumvent them are constantly evolving in bacteria and phages [80,81]. The existence of numerous ORFans that are so far unique to one branch of the T4-like phage tree [25,82] suggests that HPR might serve as a breeding ground for *de novo* phage gene birth [26,83]. The present work, as well as the recent characterization of ORFans *55*.*1* [29] and *39*.*2* [84], fit well with a third hypothesis: T4 ORFans could modify host protein functions to fine tune bacterial metabolism and permit optimal virion production (see also the “molecular splint” hypothesis in [26]). Gp39.2 shifts the open/close equilibrium of the host GroEL chaperone. Gp55.1 impacts on host folate metabolism and blocks nucleotide excision repair by interacting with FolD and UvrA respectively, and gp55.2 affects the host control of DNA supercoiling. Although deletion mutants of these three genes are perfectly viable on many *E*. *coli* K12 strains, Gp39.2 permits T4 growth on several *E*. *coli groEL* mutants that would otherwise not yield productive infections. Furthermore, we demonstrated that *55*.*2* and *55*.*1* mutants (Fig 5B and data not shown) have a detectably reduced fitness. We conclude that T4 ORFans might have a major evolutionary role by providing small but evolutionary significant growth advantages in non-optimal hosts or environmental conditions.

## Supporting Information

S1 Fig Supplementary growth curves and viability assays. (TIFF)

S2 Fig Supplementary EMSA. (TIFF)

S3 Fig Supplementary topoisomerase assays. (TIFF)

S4 Fig Supplementary analyses of *in vivo* DNA supercoiling and plasmid copy number. (TIFF)

S5 Fig Supplementary analyses of intracellular phage accumulation. (TIFF)

S1 File Supporting Materials and Methods. Construction of *E*. *coli* strains, T4 phage strains, plasmids, and genomic library; methods used for supporting figures; and list of the plasmids used in this study.
(DOCX)
