# Supplementary figures and images for: 55.2, a Phage T4 ORFan Gene, Encodes an Inhibitor of Escherichia coli Topoisomerase I and Increases Phage Fitness
Source: PLoS One. 2015 Apr 14;10(4):e0124309. doi: 10.1371/journal.pone.0124309 (PMC4396842; doi:10.1371/journal.pone.0124309)

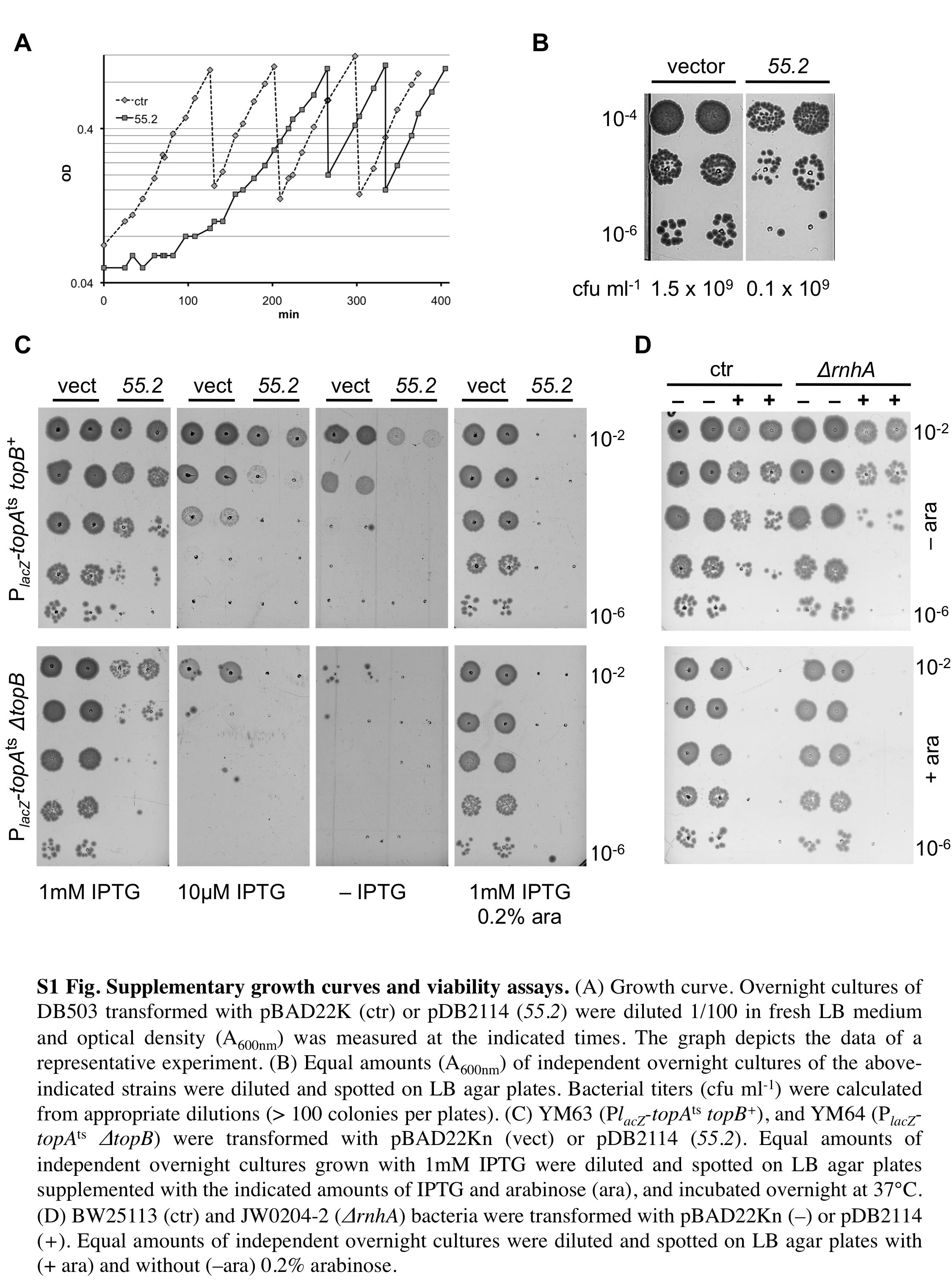

Supplement: S1 Fig — (TIFF) [file pone.0124309.s001.tiff]

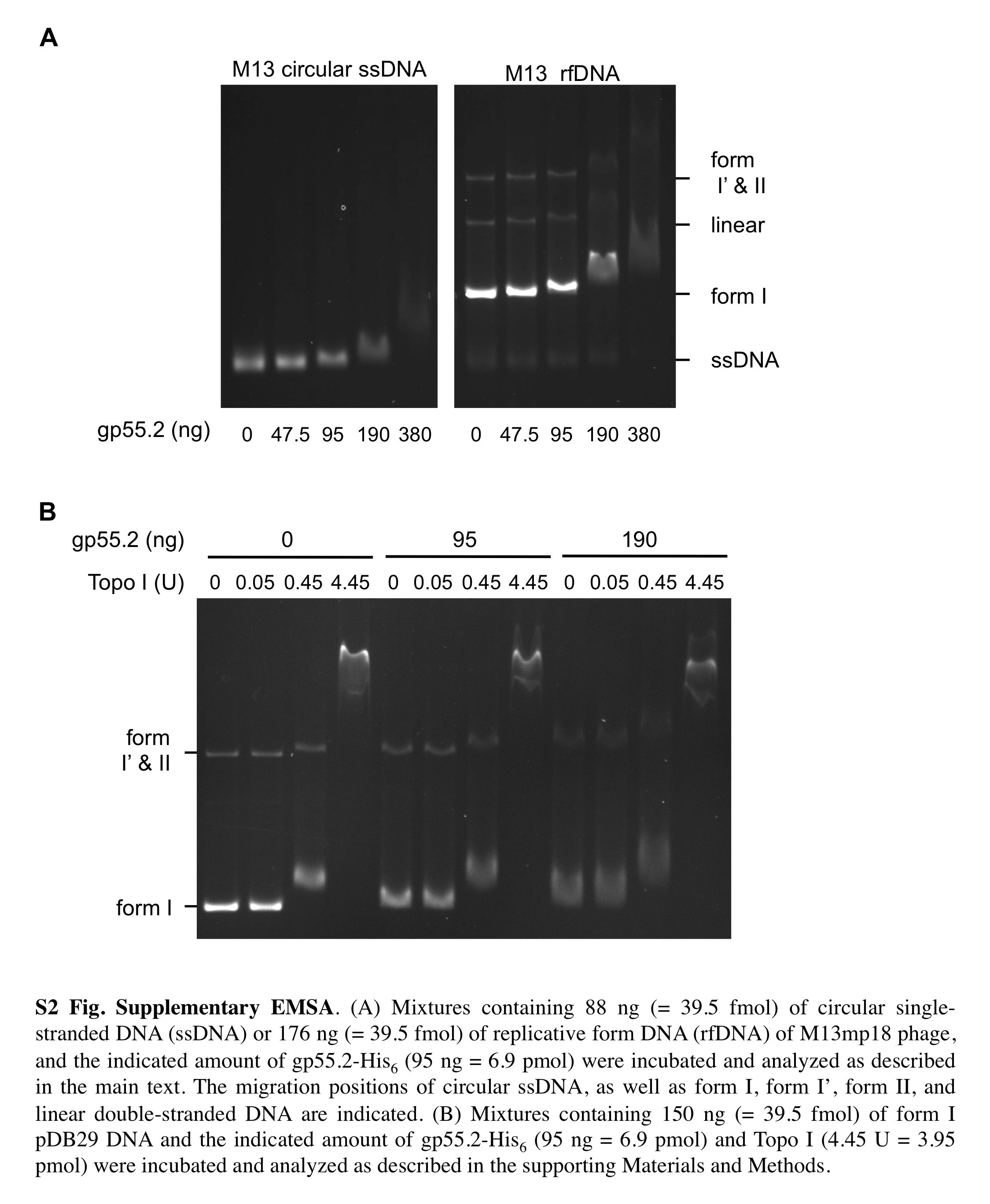

Supplement: S2 Fig — (TIFF) [file pone.0124309.s002.tiff]

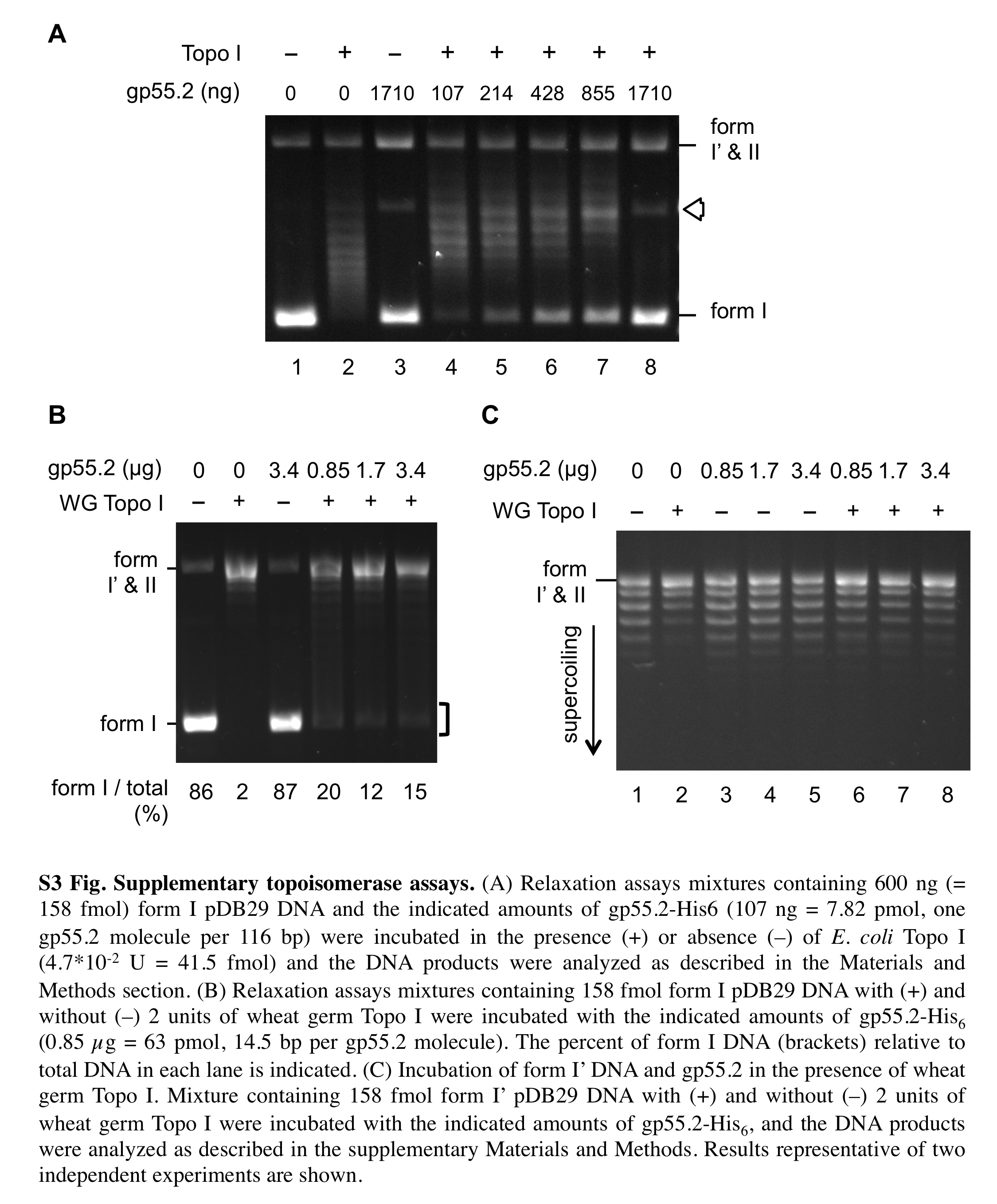

Supplement: S3 Fig — (TIFF) [file pone.0124309.s003.tiff]

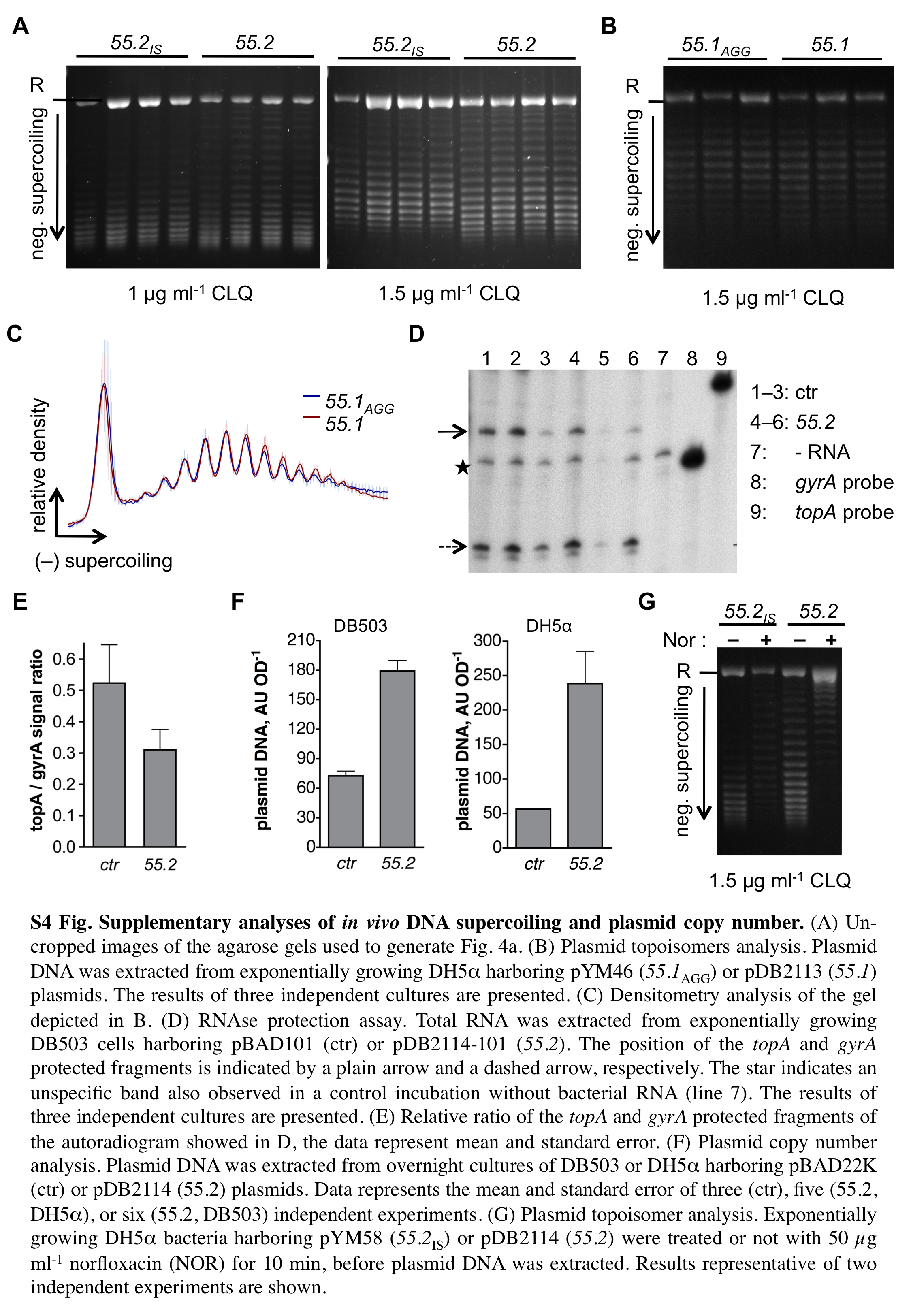

Supplement: S4 Fig — (TIFF) [file pone.0124309.s004.tiff]

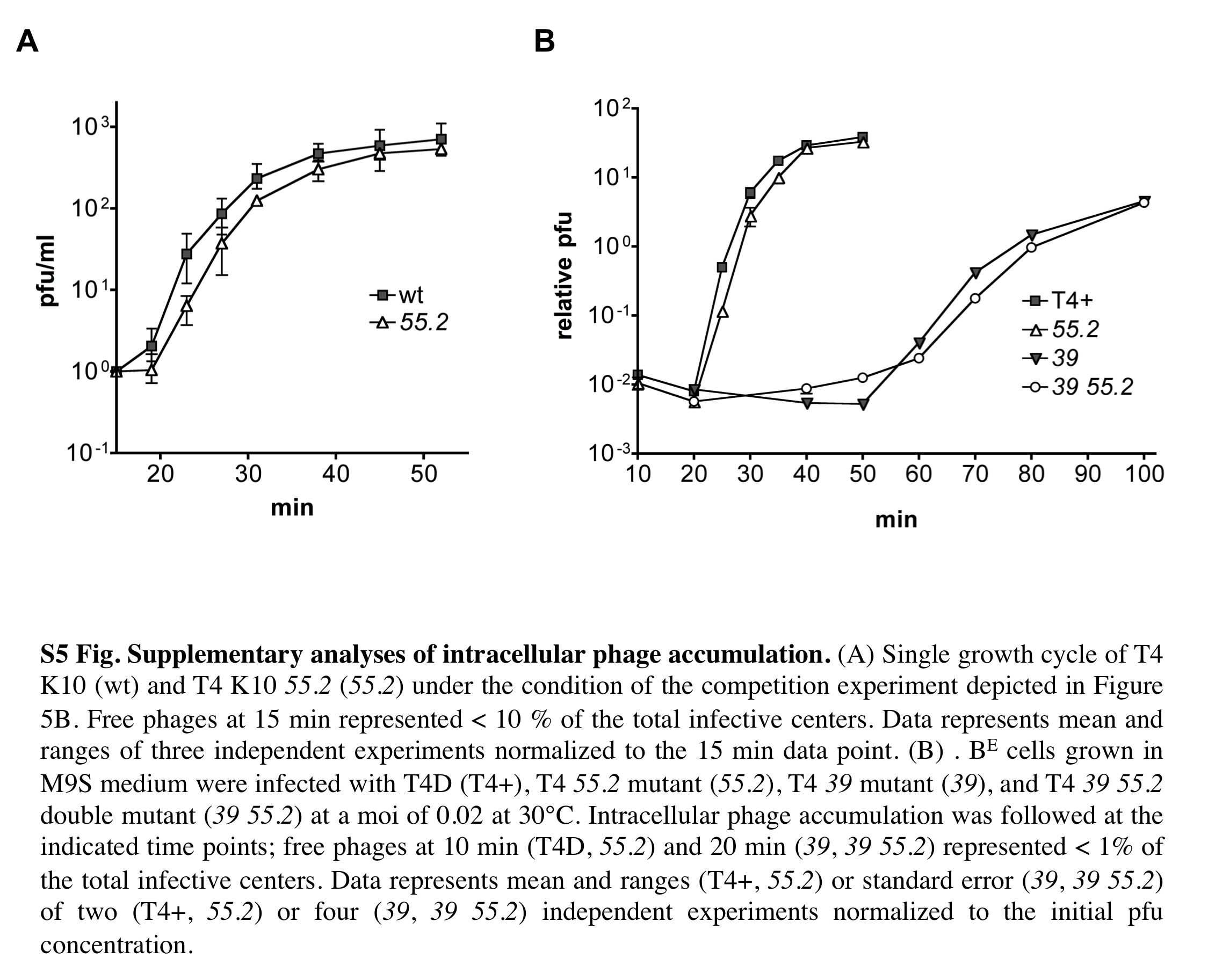

Supplement: S5 Fig — (TIFF) [file pone.0124309.s005.tiff]
