# Supplementary material for: 55.2, a Phage T4 ORFan Gene, Encodes an Inhibitor of Escherichia coli Topoisomerase I and Increases Phage Fitness
Source: PLoS One. 2015 Apr 14;10(4):e0124309. doi: 10.1371/journal.pone.0124309 (PMC4396842; doi:10.1371/journal.pone.0124309)
Supplement: S1 File — Construction of E. coli strains, T4 phage strains, plasmids, and genomic library; methods used for supporting figures; and list of the plasmids used in this study. (DOCX) [file pone.0124309.s006.docx]

**Supporting Materials and methods**

***E.coli strain construction.*** DB870 was constructed in three steps. First, CAG12131 (MG1655 *leuO::*Tn*10kan*) [[1](#_ENREF_1)] was transduced by phage P1*vir*-mediated transduction [[2](#_ENREF_2)] with a LMG194 (*Δara714* *leu::*Tn*10*) [[3](#_ENREF_3)] lysate and Tc^r^ recombinants were screened on MacConkey arabinose agar for the Ara^–^ phenotype conferred by the *Δara714* deletion. Second, this strain was transduced with a MC4100 lysate and Tc^s^ Ara^–^ recombinants were screened among Leu^+^ recombinants selected on M9 minimal glucose plates. Third, a *ydeA::kan* null mutation was transduced from DB524-1 [[4](#_ENREF_4)]. YM63 and YM64 were constructed by transducing BMW25113 and JW1752-1 (*ΔtopB::kan*) [[5](#_ENREF_5)], respectively, with a VS105 (P*_lacZ_-topA*(ts) *zci::cat*) [[6](#_ENREF_6)] lysate and selecting for Cm^r^ recombinant on plates containing 1mM IPTG.

***T4 strain construction.*** A *55.2* null mutant (ATG -> ACA mutation at T4 coordinates 40'784-40'783 that destroys the *55.2* initiation codon and generates a BsrGI site) was constructed using the T4 I/S system [[7](#_ENREF_7),[8](#_ENREF_8)]. A 668 bp fragment with the full-length *55.3* and *55.2* genes, and containing the above-described mutation was generated by PCR site-directed mutagenesis [[9](#_ENREF_9)] using two internal primers 5'-gaggataatgtaca actattcaaattaaaaac and 5'-gtttttaatttgaatagttgtacattatcctc (mutations underlined). The fragment was cloned between the EcoRI and SalI site of pBSPL0+ and the construction was verified by sequencing. The mutation carried by the recombinant plasmid was then transferred to the T4 K10 genome. The presence of the mutation was verified by PCR followed by BsrGI digestion, as well as by sequencing of the *55.3-55.1* region. To ensure that all observed phenotypes of the T4 K10-*55.2* strain were due to the *55.2* mutation, all experiments were down with two independently isolated clones. In a strain harboring a pBAD22K vector expressing *55.2*, the ATG -> ACA mutation completely abolished all *55.2* induced phenotypes, including the Ara^S^ phenotype. To generate the T4D *55.2* mutant, T4 K10-*55.2* was crossed with T4D in *E. coli* CR63 (moi of 5 for each phage) and the progeny was plated on DB503(λ) (a λ lysogen derived from DB503) to select against the *38am*, *51am*, and *rIIPT8* (*denB*-*rII* deletion) mutations present in the K10 background. Individual plaques were tested by PCR and restriction digestion to identify *55.2* mutants. The *denA*^+^ genotype of the chosen *55.2* mutant was confirmed by sequencing.

The T4D *39amEA29* was from R. H. Epstein’s collection. We identified the position of the amber codon (W39stop) by sequencing. Doing so, we found that the T4D strain used in this study has 6 nucleotide changes (G978T, A979G, T980A, C982T, G983T, and A1064C) resulting in 4 amino acid changes (L326F, I327D, R328S, and D355A) compared with the DNA sequence of gene *39* found in the complete genome of phage T4 (GenBank: AF158101.6). Yet, the protein sequences of gp39 in another sequenced T4 phage (T4T, ENA: HM137666.1) and of gp39 homologues in 15 phages closely related to T4 match our version of gp39. Therefore, we conclude that our T4D strain has a wild-type gene *39*. To generate the T4D *39am* *55.2* double mutant, T4D *39am* was crossed with T4D *55.2* in *E. coli* CR63 (moi of 5 for each phage) and the progeny was plated on CR63. Individual plaques were screened by PCR and restriction digestion to identify *55.2* mutants and by spotting on an *E. coli* B^E^ lawn at 25°C to test for the cold-sensitive phenotype caused by gene *39* mutations [[10](#_ENREF_10)]. *E. coli* B^E^ is a prototrophic *E. coli* B strain [[11](#_ENREF_11)].

***Plasmid construction.*** pDB868-2 is derived from a spontaneous mutant of pBAD33 that has a 2-fold increase in copy number (Bieler and Belin *unpublished* ). It was constructed by ligating an AvaI/XbaI-digested Klenow fragment filled-in insert of pACYC184 that contains the *tet* gene into Tth111I/ClaI-digested, Klenow fragment filled-in pBAD33 mutant.

pDB2114-33-K was constructed by ligating an EcoRI-digested, Klenow filled-in fragment of pUC4K (GE Healthcare Life Science), which contain the Kn^r^ cassette, into the unique, Klenow blunted, NcoI site of pDB2114-33. pDB2114-101 was derived from its parent plasmid by ligating the EcoRV/XbaI-digested fragment with the EcoRV/XbaI-digested pBAD101 vector. pMCN1 expressing *55.2* with a C-terminal His_6_ tag was generated by PCR amplification of pDB2114 using the following primer pairs: 5'-cctgacgtttttatcgc / 5'-CAGTCTAGATTAATGGTGA TGGTGATGGTGACCACCTACCTTAGTATTTTTCAG (2x Gly linker and His_6_ sequence underlined) and cloning in the EcoRI and XbaI sites of pBAD22Kn.

pYM46 was constructed by PCR amplification of pDB2113 using the following primer pair: 5'-AGGAATTCACCAGGAACGAAGC / 5'- CTCATCCGCCAAAACA (mutation underlined) and cloning in the EcoRI and XbaI sites of pBAD22Kn. This replaced the ATG start codon of 55.1 by AGG and completely abolished all *55.1* induced phenotypes. pYM58 was constructed in two steps. First the PCR insert used to generate the T4 K10-*55.2* phage mutant (cf. above) was cloned into the XbaI site of pBAD22K. Second, this plasmid was digested with BsrGI and NcoI, filled in with Klenow fragment and self-ligated. The resulting plasmid, pYM58, is identical to pDB2114, except for a 5bp insertion that destroys the *55.2* open reading frame just after the ATG start codon; this insertion completely abolished the *55.2*-induced Ara^S^ phenotype.

pDB34 was the original *55.2* suppressor plasmid isolated from the *E. coli* library, its insert covers position 1’328’355 to 1’332'614 on the *E. coli* K12 MG1655 genome (coordinates based on www.ecocyc.org). pDB34-8 was obtained by digesting pDB34 with SmaI and HindIII, filling in with Klenow fragment and self-ligation. pDB34-8-4 was constructed by digesting pDB34-8 with SphI and self-ligation, the absence of mutation in the *topA* coding and promoter sequences was verified by sequencing.

pYM61 was generated by digesting pDB34-8-4 with EcoRI and BamHI, and cloning a 368 bp fragment (containing position 2093 to 2460 of gene *topA*) into the EcoRI and BamHI sites of pBluescript II SK+ (Stratagene). pYM62 was constructed by amplifying position 336 to 456 of gene *gyrA* using the following primer pair: 5'-GTAGAATTCCGACGGCGACTC / 5'-GTAGGATCCCGTGCCGTC ATAG and cloning into the EcoRI and BamHI sites of pBluescript II SK+. All PCR generated constructions were verified by sequencing.

***Genomic library.*** An *E. coli* genomic DNA library was constructed by partial digestion of DB870 DNA with Sau3AI and ligation of the 4-6 kb fragments in the BamHI site of pDB868-2.

***Relaxation assays and assembly of gp55.2 and DNA in the presence of wheat germ Topoisomerase I.*** Wheat germ topoisomerase was purchased from Promega (#M285) and reactions were performed in 1x WG Topo I buffer (50mM Tris pH 7.5, 50mM NaCl, 0.1mM EDTA, 1mM DTT). For relaxation assays, form I of pDB29 DNA (0.6 µg per reaction = 158 fmol) was first incubated with the indicated amounts of gp55.2-His_6_ for 15 min at 37°C, then the indicated amounts of Topo I were added and reactions were incubated at 37°C for 30 min. Reactions were terminated and analyzed as explain in the main Materials and Methods section. To check for the potential introduction of superhelical turn by gp55.2, form I’ pDB29 DNA was used instead of form I DNA.

***EMSA***. To determine if *E. coli* Topo I and gp55.2 can bind the same DNA molecule, EMSA reaction mixture were assembled on ice in the following order: plasmid DNA diluted in the reaction buffer, gp55.2, and Topo I. The rest of the EMSA was performed as described in the main Materials and Methods section.

***RNA extraction and RNAse protection assay.*** Cells were grown in LB media with aeration at 37 °C to *A*_600 nm_ = 0.5. RNA was extracted with Trizol (Life Technologies) according to manufacturer instruction. Sample concentration was determined with a UV NanoDrop spectrophotometer (NanoDrop products Wilmington, DE) while RNA quality was checked by agarose electrophoresis. ^32^P-labeled RNA probes were generated with T3 RNA polymerase on pYM61 plasmid linearized with SnaBI for *topA* and on pYM62 linearized with EcoRI for *gyrA*. RNAse protection assays were performed as previously described [[12](#_ENREF_12)] with 2 μg of total RNA and both probes. The relative ratio of the protected RNA fragments was determined by filmless autoradiography using a Typhoon FLA 7000 scanner (GE Healthcare) followed by densitometry analysis using the ImageQuant software (GE Healthcare).

***Preparation of single-stranded and replicative form of bacteriophage M13mp18 DNA.*** Single-stranded M13mp18 DNA was purchased from USB corporation (70704, USB, Affymetrix), transformed into XL1-Blue competent cells, and plated on a top agar lawn of XL1-Blue bacteria. An individual plaque was used to prepare a phage stock and replicative form DNA was purified according to published protocol [[13](#_ENREF_13)].

**TABLE S1. Plasmids**

| **Plasmid** | **Description** | **Reference** |
| --- | --- | --- |
| pAST111 | pRC7 expressing *topA* from its promoters (includes 150bp upstream of *topA* start codon), LacI^-^ | [[14](#_ENREF_14)] |
| pBAD101 | Vector with pSC101 replication origin, P_BAD_ promoter and *araC*, Sp^r^ | [[15](#_ENREF_15)] |
| pBAD22 | Vector with pBR322 replication origin, P_BAD_ promoter and *araC*, Ap^r^ | [[3](#_ENREF_3)] |
| pBAD22K | pBAD22 with additional Kn^r^ | [[16](#_ENREF_16)] |
| pBAD33 | Vector with pACYC184 replication origin, P_BAD_ and *araC*, Cm^r^ | [[3](#_ENREF_3)] |
| pBAD33-K | Vector with pACYC184 replication origin, P_BAD_ and *araC*, Kn^r^ | [[16](#_ENREF_16)] |
| pDB29 | pBR322 with a 1391 bp mouse urokinase cDNA insert into the PstI site | [[17](#_ENREF_17)] |
| pDB2113 | pBAD22Kn expressing *55.1* from P_BAD_ | [[16](#_ENREF_16)] |
| pDB2114 | pBAD22Kn expressing *55.2* from P_BAD_ | [[16](#_ENREF_16)] |
| pDB2114-33 | pBAD33 expressing *55.2* from P_BAD_ | [[16](#_ENREF_16)] |
| pDB2114-33-K | pBAD33-K expressing *55.2* from P_BAD_ | this study |
| pDB2114-101 | pBAD101 expressing *55.2* from P_BAD_ | this study |
| pDB34-8 | pDB868-2 expressing *topA* and *yciN* (includes the full *topA* and *yciN* promoters region), Cm^r^, Tet^s^ | this study |
| pDB34-8-4 | pDB868-2 expressing *topA* from its promoters (includes 275bp upstream of *topA* start codon), Cm^r^, Tet^s^ | this study |
| pDB868-2 | Vector with a mutated pACYC184 replication origin (copy number increased by 2 fold), Cm^r^, Tet^r^ | this study |
| pMCN1 | pBAD22K expressing *55.2*-His_6_ (C-terminal tag) from P_BAD_ | this study |
| pRC7 | Unstable mini-F plasmid, *lacI^Q^ZYA*, Ap^r^ | [[18](#_ENREF_18)] |
| pYM46 | pDB2113 with an ATG ->AGG mutation of the start codon | this study |
| pYM58 | pDB2114 with a 5 bp insertion after the ATG start codon that generates a new unrelated 18 amino acid ORF | this study |
| pYM61 | pBluescript II SK with an EcoRI, BamHI fragment containing part of gene *topA* | this study |
| pYM62 | pBluescript II SK with a PCR fragment containing part of gene *gyrA* | this study |

**References**

1. Singer M, Baker TA, Schnitzler G, Deischel SM, Goel M, et al. (1989) A collection of strains containing genetically linked alternating antibiotic resistance elements for genetic mapping of *Escherichia coli*. MicrobiolRev 53: 1-24.

2. Miller JH (1992) A short course in bacterial genetics. Cold Spring Harbor, NY: Cold Spring Harbor Laboratory Press.

3. Guzman LM, Belin D, Carson MJ, Beckwith J (1995) Tight regulation, modulation, and high-level expression by vectors containing the arabinose P_BAD_ promoter. J Bacteriol 177: 4121-4130.

4. Bost S, Silva F, Belin D (1999) Transcriptional activation of *ydeA*, which encodes a member of the major facilitator superfamily, interferes with arabinose accumulation and induction of the *Escherichia coli* arabinose P_BAD_ promoter. J Bacteriol 181: 2185-2191.

5. Baba T, Ara T, Hasegawa M, Takai Y, Okumura Y, et al. (2006) Construction of Escherichia coli K-12 in-frame, single-gene knockout mutants: the Keio collection. Mol Syst Biol 2: 2006 0008.

6. Stupina VA, Wang JC (2005) Viability of *Escherichia coli* *topA* mutants lacking DNA topoisomerase I. J Biol Chem 280: 355-360.

7. Kreuzer KN, Selick HE (1994) Directed insertion/substitution mutagenesis. In: Karam JD, editor. Molecular biology of bacteriophage T4. Washington, D. C.: ASM press. pp. 452-454.

8. Selick HE, Kreuzer KN, Alberts BM (1988) The bacteriophage T4 insertion/substitution vector system. A method for introducing site-specific mutations into the virus chromosome. J Biol Chem 263: 11336-11347.

9. Ho SN, Hunt HD, Horton RM, Pullen JK, Pease LR (1989) Site-directed mutagenesis by overlap extension using the polymerase chain reaction. Gene 77: 51-59.

10. Mufti S, Bernstein H (1974) The DNA-delay mutants of bacteriophage T4. J Virol 14: 860-871.

11. Daegelen P, Studier FW, Lenski RE, Cure S, Kim JF (2009) Tracing ancestors and relatives of *Escherichia coli* B, and the derivation of B strains REL606 and BL21(DE3). J Mol Biol 394: 634-643.

12. Belin D (1997) The use of RNA probes for the analysis of gene expression. MolBiotechnol 7: 153-163.

13. Sambrook J, Fritsch EF, Maniatis T (1990) Molecular cloning. A laboratory manual.: Cold Spring Harbor Laboratory Press.

14. Stockum A, Lloyd RG, Rudolph CJ (2012) On the viability of *Escherichia coli* cells lacking DNA topoisomerase I. BMC Microbiol 12: 26.

15. Bieler S, Silva F, Soto C, Belin D (2006) Bactericidal activity of both secreted and nonsecreted microcin E492 requires the mannose permease. J Bacteriol 188: 7049-7061.

16. Mattenberger Y, Mattson S, Metrailler J, Silva F, Belin D (2011) *55.1*, a gene of unknown function of phage T4, impacts on *Escherichia coli* folate metabolism and blocks DNA repair by the NER. Mol Microbiol 82: 1406-1421.

17. Belin D, Vassalli J-D, Combepine C, Godeau F, Nagamine Y, et al. (1985) Cloning, nucleotide sequencing and expression of cDNAs encoding mouse urokinase-type plasminogen activator. EurJBiochem 148: 225-232.

18. de Boer PA, Crossley RE, Rothfield LI (1989) A division inhibitor and a topological specificity factor coded for by the minicell locus determine proper placement of the division septum in *E. coli*. Cell 56: 641-649.
